# Guiding the humoral response against HIV-1 toward a MPER adjacent region by immunization with a VLP-formulated antibody-selected envelope variant

**DOI:** 10.1371/journal.pone.0208345

**Published:** 2018-12-19

**Authors:** Carolina Beltran-Pavez, Carolina B. Ferreira, Alberto Merino-Mansilla, Amanda Fabra-Garcia, Maria Casadella, Marc Noguera-Julian, Roger Paredes, Alex Olvera, Isabel Haro, Christian Brander, Felipe Garcia, Jose M. Gatell, Eloisa Yuste, Victor Sanchez-Merino

**Affiliations:** 1 AIDS Research Unit, Institut d’Investigacions Biomediques August Pi i Sunyer, Barcelona, Spain; 2 HIVACAT, Barcelona, Spain; 3 IrsiCaixa AIDS Research Institute, Hospital Germans Trias i Pujol, Badalona, Barcelona, Spain; 4 Universitat de Vic-Universitat Central de Catalunya (UVic-UCC), Vic, Spain; 5 Unit of Synthesis and Biomedical Applications of Peptides, IQAC-CSIC, Barcelona, Spain; 6 ICREA, Pg. Lluís Companys 23, 08010 Barcelona, Spain; 7 Infectious Diseases Unit, Hospital Clinic, Barcelona, Spain; Boston College, UNITED STATES

## Abstract

Preventive HIV-1 vaccine strategies rely on the elicitation of broadly neutralizing antibody (bNAb) responses, but their induction *in vivo* by vaccination remains challenging. Considering that the ability of an epitope to elicit effective humoral immunity depends on its exposure on the virion, we have used a reverse genetics approach to select variants from an HIV-1 AC10_29 randomly mutated envelope library that showed increased affinity for a selected bNAb (4E10 bNAb targeting the HIV-1 MPER region). Isolated envelope sequences were analyzed by deep-sequencing showing a small number of dominant changes, including the loss of four potential N-linked glycosylation sites and disruption of the V1/V2 loop. Accordingly, the dominant variant (LR1-C1), showed not only increased affinity for MPER bNAbs 4E10 and 2F5, but also higher affinity for an additional antibody targeting the V3 loop (447-52D) that could be a consequence of an open conformation tier 1-like Env. Furthermore, the amino acids specific for the selected variant are associated with an increased sensitivity for 4E10 and 2F5 antibodies. *In vivo* studies showed that sera from mice immunized with LR1-C1 viruses possessed an improved neutralizing activity compared to the wild-type AC10_29 env. While Virus Like Particles (VLPs) carrying this envelope were unable to induce detectable neutralizing activity in immunized rabbits, one animal showed antibody response to the 4E10-proximal region. Our data establish a novel approach that has the potential to yield HIV envelope immunogen sequences that direct antibody responses to specific envelope regions.

## Introduction

Many classical vaccine approaches are based on the induction of neutralizing antibodies to surface antigens which often are the most variable portions of the pathogen. In HIV-1 infection, the viral envelope glycoprotein (Env) is the sole virus-specific target of neutralizing antibodies [1–3]. However, Env has evolved several mechanisms to evade or minimize neutralization such as reduced expression on the viral surface, high variability intra and inter-subtypes, glycan coating and steric occlusion [4]. Although these evasion strategies present a major challenge for the induction of neutralizing antibodies by vaccination, it is remarkable that 10–25% of chronically HIV-1 infected individuals show high titers of broadly neutralizing antibodies (bNAbs) with broad neutralizing capacity even against different HIV-1 clades [5–7]. Moreover, it has been reported that up to 50% of HIV-1 infected individuals are able to develop significant plasma neutralization breadth over several years of infection [8]. These studies suggest that the induction of such antibodies generally requires long periods of antigen exposure, which may be a major obstacle for HIV vaccine development. However, work by our lab and by others have described that bNAbs, albeit rare, can be induced much earlier than previously thought, even within the first months of HIV-1 infection [9,10]. Importantly, passive transfer of such bNAbs to humanized mice and monkeys effectively protects them against HIV and chimeric simian-human immunodeficiency virus (SHIV) infection, respectively [11–18]. Furthermore, passively transferred bNAbs have also shown efficacy in controlling viral replication in HIV-1 infected individuals and support the idea that bNAbs might be useful for treatment and prevention of HIV-1 infection [19–22]. This evidence supports the hypothesis that the induction of protective levels of bNAbs by immunization is feasible, if the appropriate immunogens are provided. Thereby, a critical aspect of immunogen delivery is to ensure that the envelope protein is presented in the conformation most appropriate to induce the antibodies of interest. One possible approach to achieve this, which has been shown to be a promising strategy to induce strong immune responses in a safe manner, is immunogen delivery in the context of virus-like-particles (VLPs). VLPs have many advantages as immunogens since they are able to produce protein and lipid self-assembly to generate non-infectious particles mimicking the morphology of wild-type infectious virus. They have also the capacity of presenting native Env trimers on their surfaces and can be presented to T cells in order to mount both humoral and cellular antiviral responses. Currently, there are four VLP-based prophylactic vaccines commercially available such as Human Papilloma virus (HPV), Hepatitis B (HBV), Malaria and Influenza virus. Many others are under clinical or preclinical development supporting this strategy as a promising safe approach to induce strong immune responses [23–25].

Based on these considerations, we decided to take a radically different approach for HIV immunogen design based on the usage of randomly mutated Env libraries. This strategy has been used before for soluble envelope trimers, however, it has not been used for virion incorporated envelopes [26]. Random mutagenesis has been a powerful tool for elucidating protein structure-function relationships and for modifying proteins to improve or alter their characteristics. Sequential rounds of error-prone PCR to introduce random mutations and screening of the resultant mutant libraries have been used to enhance the catalytic activity and the thermal and oxidative stabilities of different enzymes. The approach has also been employed for the identification of drug resistant HIV-1 mutants [27–29]. In the present study, we have applied this strategy to produce and test envelope protein sequences with the potential to better present bNAb epitopes and to elicit *in vivo* humoral immune responses toward the corresponding epitopes. Our approach is based on the *in vitro* selection of envelope variants on the surface of viral particles that possess increased affinity for bNAbs using a reverse genetics approach. Using this method, we have successfully selected an envelope variant with increased affinity for the broadly neutralizing antibody 4E10 (LR1-C1) that incorporated several amino acid replacements shown to increase sensitivity to neutralization with bNAb 4E10 and 2F5. Moreover, *in vivo* immunization using VLP-based delivery induced an immune response to a region proximal to the epitope recognized by the antibody used for selection.

## Materials and methods

### Rapid Immunogen Selection (R.I.S.) method

Patent applications WO 2012113921 and 2014027082. **i) *In-vitro* random mutagenesis and subcloning.** Random mutations in the HIV *env* gene were introduced using a Genemorph II Random Mutagenesis kit (Stratagene, La Jolla, CA) following the manufacturer’s recommendations and using the AC10_29 sequence as the starting template. For mutagenesis, primers 179 and 180 were used (S1 Table). For the generation of a library of chimeric virions, randomly mutated *env* genes were inserted into pNL4-3 (obtained from the National Institutes of Health AIDS Research and Reference Reagent Program; NIH ARRRP) using convenient restriction sites (XbaI and NotI) to generate replication competent virions. Randomly mutated *env* genes were also introduced into pcDNA 3.1+ (Invitrogen; Carlsbad, CA, U.S.A.) using XbaI and NotI restriction sites. pcDNA cloning was chosen for library characterization due to its higher cloning efficiency that has, therefore, a minor cloning associated bottleneck. Five independent ligations and transformations were performed simultaneously to avoid the loss of variability and mixed together in the up scaling of amplification. Env-positive clones were sequenced with primers 183, 185, 186, 190, 192 and 193 (S1 Table). **ii) Virion library generation**. Virion library was produced by transfecting the DNA constructions in 293T cells using the calcium phosphate method according to the manufacturer´s recommended protocol (ProFection mammalian transfection system; Promega, Madison, WI). **iii) Virion capture assay**. 96-well plates were coated overnight at 4°C with 0.5μg of goat anti-human polyclonal anti-Fc antibodies in PBS. Next, wells were blocked with 3% bovine serum albumin (BSA) in PBS for 1h at 37°C. Viruses generated from the library were used for selection (20ng of p24) with 4E10 antibody (5μg/well) for 1h at 37°C with agitation (450 r.p.m.) in a round bottom 96-well plate, allowing binding to occur. Virus-Ab complexes and a control virus suspension without antibody were then transferred to blocked microtiter wells previously coated with anti-Fc antibodies. After a 2h incubation at 37°C, wells were washed six times with PBS, and RNA was extracted with High Pure Viral RNA kit by adding the binding buffer directly to the well (Roche, Basel, Switzerland). **iv) Nested RT-PCR HIV-1 *env* RNA amplification**. Isolated HIV-1 *env* RNA was then transferred to an Eppendorf tube and, next, it was amplified by reverse transcriptase polymerase chain reaction (RT-PCR). An RNA template was used for the RT-PCR reaction. RNA was reverse transcribed with primer 102 [30]) at 50°C for 20 min with GeneAmp Gold RNA PCR reagent kit (Applied Biosystems, Waltham, MA, U.S.A.). *Env* was amplified with the primers 101[30] and 104 [30] from cDNA followed by a nested PCR with primers 179 and 180 (S1 Table) and Expand High Fidelity PCR system (Roche, Basilea, Switzeland). Amplified *envs* were subcloned and virus stocks were generated as described above.

### Deep-sequencing

DNA amplification was carried out by a PCR with primers 454_1 and 454_2 for amplicon 1; 454_3 and 454_4 for amplicon 2 and 454_5 and 454_6 for amplicon 3 (S1 Table and S1 Fig). Primers were further ligated with multiple identifier adaptors (MID) specific to the Titanium sequencing chemistry (454/GS/Junior, Roche Diagnostics, Branford, CT). The amplicon library was generated in triplicate followed by pooling and purification of triplicate PCR products using magnetic beads (Agencourt AMPure Kit, Beckman Coulter Inc., Benried, DE) to eliminate primer-dimers. The number of molecules was quantified by fluorometry (Quant-iT PicoGreen dsDNA assay kit, Invitrogen Corp., Carslbad, CA, USA). Emulsion PCR (emPCR) was carried out according to the 454 Titanium emPCR protocol (Roche Diagnostics, Branford, CT). The sequencing run was performed on a picotiter plate using the 454/GS/Junior.

### Virion-antibody affinity assays

96-well plates were coated overnight at 4°C with 0.5μg of goat anti-human polyclonal anti-Fc antibodies in PBS. Next, wells were blocked with 3% bovine serum albumin (BSA) in PBS for 1h at 37°C. Serial 2-fold dilutions of recombinant viruses were incubated with the corresponding mAbs (5μg/well) for 1h at 37°C with agitation (450 r.p.m.) allowing binding to occur. The virus-Ab complexes and the control virus suspension with no mAb were then transferred to the blocked microtiter wells previously coated with the immobilized anti-Fc antibodies. Control virus suspension with no mAb showed the background level of non-specific binding. After a 2h incubation at 37°C, wells were washed six times with PBS, and virus equivalents were quantified by p24 ELISA. Statistical analysis was conducted by R using One-way NOVA followed by Newman Keuls post hoc test (***p<0.001 and *p<0.05). Data are represented as mean ± SDEV.

### Specific envelope amino acid analysis

Gp160 sequences were downloaded from CATNAP database in FASTA format (http://hiv.lanl.gov/catnap) together with IC_50_ values for each antibody-virus pair [31]. The analysis was performed with 675 antibody-virus pairs for 4E10, 676 pairs for 2F5, 144 for 447-52D, 639 for VRC01, 669 for 2G12, 423 for PG16, 255 for PGT151 and 715 for b12. HIV-1 isolates with W503 and N643 amino acids could not be included in the analysis because there was no neutralization data for these isolates in the database. IC_50_ geometric mean of undetected neutralization was set to 100μg/ml, as described at CATNAP database. Statistical significance was evaluated with a Mann-Whitney U test. Simple comparisons were made with the use of a two-sided alpha level of 0.05.

### Optimization of Gag sequence as a T-cell immunogen

An artificial full-length Gag sequence, referred to as *dGag* was used for the formation of VLP structures. The dGag sequence is based on the 2001 HIV-1 clade B Gag consensus sequence available at the Los Alamos National Laboratory HIV database with modifications at 32 amino acid positions. These sequence changes were introduced to complement our in-house developed T cell immunogen sequence [32] at positions that are frequent targets of the virus-specific CD8 T cell responses (HIV database: https://www.hiv.lanl.gov; [33]) and include residues with more than 10% frequency in the clade B sequence alignment. No changes were introduced in the oligomerization region in p24.

### Generation of virus-like particles

HIV-1-dGag-VLPs, composed of the HIV-1 dGag sequence, were produced by transient transfection with the FreeStyle 293 Expression System (Invitrogen, Carlsbad, CA, USA) co-transfecting with a pcDNA-dGag plasmid and codon optimized pcDNA3.1-AC10_29 or pcDNA3.1-LR1C1 envelope expression plasmids provided by GeneArt Gene Synthesis (ThermoFisher Scientific, Waltham, MA, USA). For transfection, 1μg DNA/10^6^cells of each HIV-1 dGag or HIV-1 Env-expression plasmids were used. Different ratios of dGag/Env expression plasmids were tested and 3:1 and 10:1 ratios were chosen for AC10_29 and LR1-C1 VLPs respectively in order to obtain similar Env incorporation for each VLP. At 48 hours post-transfection, VLPs were harvested by ultracentrifugation [34]. Briefly, VLP supernatants were first clarified through centrifugation twice at 800g for 5 min and then ultracentrifugated at 50000g for 32 min. Pellets were ultracentrifuged once more at 160000g for 10 min and resuspended in Trehalose 5%/PBS to preserve VLP integrity and stored at -80°C [35].

### Immuno-electron cryo-microscopy

VLPs were produced and ultracentrifugated as described above. VLP pellets were fixed with a 4% paraformaldehyde 0.1% glutaraldehyde solution in 0.1M PB for 1h at 4°C. Next, the solution was removed carefully and pellets were stored at 4°C in a 2% paraformaldehyde in 0.1M PHEM at 37°C and cryo-preserved in 2.3M sucrose in 0.1M PHEM at 4°C overnight. Then, they were mounted in pins and frozen in liquid nitrogen. Ultrathin sections were obtained in a FC7 ultramicrotome (Leica Microsystems, Wetzlar, Germany) and were immunolabeled with an anti-gp120 antibody (447-52D; Polymun Scientific, Klosterneuburg, Austria) and a gold-conjugated anti-human antibody (Jackson Immunoresearch, West Frove, PA, USA). Samples were visualized in a Transmission Electron Microscopy (TEM) Tecnai Spirit (FEI) at 120 kV with a LaB6 cathode and equipped with a 1kx1k Megaview CCD Camera.

### Western blotting

Env incorporation per virion or per VLP was analysed by western blotting in SDS-PAGE gels. Identical quantities of p24 or p55 were mixed with Laemmli buffer supplemented with 5% β-mercaptoethanol, boiled for 10 minutes and kept on ice. Env expression in 293T cells was also analysed by western blotting after lysing with a Cell lysis buffer (Cell Signaling Tecnology, Danvers, MA, U.S.A) and boiled for 10 minutes. Samples were then electrophoresed at 160V for 90 minutes through a 4 to 12% polyacrylamide-sodium dodecyl sulfate gradient gel (GE Healthcare, Chicago, IL, USA). Following electrophoresis, proteins were transferred in a semi-dry system onto a PVDF membrane for 45 minutes at 0,1mA. Membranes were blocked with 5% skim milk in Tris-buffered saline tween 20 (TBST) for 1h at room temperature. Membranes were then incubated with antibodies recognizing the gp120 (447-52D provided by the NIH AIDS Research and Reference Reagent Program), Gag (p24 and p55) [p24 Antibody (24–4); Santa Cruz Biotechnology, Santa Cruz, CA, USA] and β-actin [β-Actin (13E5) Rabbit mAb; Cell Signaling Technology, Danvers, MA, U.S.A.). A goat anti-human-HRP (SC-2907; Santa Cruz Biotechnology, Santa Cruz, CA, USA) was used to detect gp120. Finally, Env and Gag proteins were detected with Clarity Western ECL (Bio-rad, Hercules, CA, USA) using the ImageQuant LAS 500 imager (GE Healthcare, Chicago, IL, USA). Quantitative western blot was performed as described previously [36] with a standard curve of p24 recombinant protein (HIV-1/NL43 p24 capsid p1M) to quantify p55 in VLP samples. The presence of trimers was analysed by western blotting in BN-PAGE gels as described previously [37]. VLPs were solubilized at room temperature for 10 min in 0.12% Triton X-100 in 1mM EDTA-1.5 aminocaprotic acid with 1μl of protease inhibitor cocktail (Sigma-Aldrich, St. Louis, MO, USA) at 1:1 ratio. The sample buffer used was 100mM MOPS, 100mM Tris-HCl pH: 7.7, 40% glycerol and 0.1% coomassie Blue G250 added at 1:1 ratio. Samples were loaded onto 4–12% Bis-Tris NuPAGE (Invitrogen, Carlsbad, CA, USA) separated for 3h at 100V at 4°C using High Molecular Weight calibration kit for native electrophoresis (Amersham, Buckinghamshire, UK) as a size standard. Proteins were blotted in a wet tank onto a PDF membrane at 4°C and 30V overnight. Then, membranes were blocked with 4% nonfat milk/PBS buffer for 1 hour at room temperature and stained with an anti-gp120 and anti-gp41 antibody cocktail (MAbs 2G12, b12, 39F, 2F5, 4E10 and 447-52D at 1μg/ml each one) overnight at 4°C. Finally, membranes were washed 3 times with PBS, probed by an anti-human-HRP (Jackson ImmunoResearch, West Grove, CA, USA) and proteins were detected by Pierce ECL Western Blotting Substrate (Thermo Fisher Scientific, Waltham, MA, USA).

### Immunization of BALB/c mice

The viral stocks used for immunization were obtained by transient transfection in 293T cells as described above. Viral stocks were inactivated by incubation with Aldritiol-2 1mM at 37°C for 2h under continuous agitation. Female BALB/c mice aged 6 to 8 weeks and specific pathogen free (SPF) were utilized. Mice were fed *ad libitum* with a standard diet and kept under a light-dark cycle of 12h. Each group was composed of 5 animals. Both groups were inoculated with 100μl of AT-2 inactivated virus (2.5μg/ml p24 quantified as explained above) and 100μl of complete Freund adjuvant subcutaneously divided among all 4 legs (t = 0). Two weeks later (t = 14 days), mice were inoculated with 100μl of AT-2 inactivated virus (2.5μg/ml p24) and 100μl of incomplete Freund adjuvant subcutaneously into each leg. After two weeks (t = 28 days), between 400μl and 1ml of blood were extracted by cardiac puncture under isoflurane anesthesia from both groups. Mice euthanasia was performed by overdose of isoflurane. One subject from the first group perished before conclusion of the four weeks for reasons not related to the experimental protocol (S4 Fig). These experiments were performed in a BSL3 Biocontainment Facility at Centre de Recerca en Sanitat Animal (CReSA) with the support by CReSA staff (Barcelona, Spain).

### Rabbit immunizations

5 groups of rabbits were immunized (4 per group): HIV-1-Gag-VLPs as a control group (G1); HIV-1-Gag-VLPs with AC10_29 Env (G2/G4) and LR1-C1 Env (G3/G5). In G1-G3 groups Adjuplex adjuvant (Sigma-Aldrich, St. Louis, MO, USA) was used while no adjuvant was added in groups G4 and G5. The vaccination protocol used was a DNA prime followed by one DNA boost (500μg of DNA/boost/animal) and 3 VLP boosts (20μg of p24 of VLP/boost; S5 Fig). 15 weeks after immunization, the presence of anti-HIV-1 antibodies was analyzed using New Lav Blot I kit (Bio-Rad, Hercules, CA, USA) substituting the horseradich peroxidase-conjugated for a alkaline phosphatase-goat anti-rabbit IgG (Invitrogen, Carlsbad, CA, USA) diluted 1/1,000 in 5% milk in PBS. All rabbits were maintained in 785 x 840 x 1840 h (mm) cages with access to water and food ad libitum and under constant humidity and temperature with a light/dark cycle of 12 hours. All procedures were conducted in accordance with the guide-lines established by the University of Barcelona´s Bioethics Committee, as stated in Law 214/97 (July 30^th^) drawn up by the Generalitat de Catalunya.

### Enzyme-linked immunoabsorbent assays (ELISA)

Wells of 96-well high-binding plates (Inmulon, ThermoFisher Scientific, Waltham, MA, USA) were coated with 100μl of AC10_29 (HIV-1, clade B; gp120; Abnova, Tipei, Taiwan) at 200ng/well with phosphate-buffered saline (PBS) and incubated at 4°C overnight. Next, wells were blocked with 150μl of 5% nonfat powdered milk in PBS at 37°c for 1h. 100μl of the corresponding sera dilutions in 5% PBS-milk were added to each well and incubated at 37°C for 1h. After washing three times with PBS plus 0.05% Tween 20, 100μl of horseradich peroxidase-conjugated goat anti-rabbit immunoglobulin G antibody (sc-2004, Santa Cruz Biotechnology, Santa Cruz, CA, USA) diluted 1/2,000 in 5% milk in PBS was added to each well, and the plates were incubated at 37°C for 1h. Plates were then washed 6 times with PBS-Tween, and 100μl of tetramethylbenzidine reagent (Calbiochem, San Diego, CA, USA) was added to each well. 10 minutes later, 50μl of hydrochloric acid was added to each well, and optical density at 450nm was measured using a spectrophotometer (Sunrise, TECAN, Zürich, Switzerland). For Env peptide ELISA, 15-mer peptides corresponding to the V3 loop and the gp41 ectodomain of AC10_29 Env sequence (283 to 341 amino acids and 629 to 686 respectively, numbered according to HXB2) were synthesized by Bionova (V3 peptides; Bionova, Halifax, Canada) and by the Unit of Synthesis and Biomedical Applications of Peptides, IQAC-CSIC, Barcelona (gp41 peptides). The assay was performed as described previously [38].

### Neutralization assays

400 μl of sera from immunized mice and rabbits were pre-adsorbed with 1x10^6^ cells that were used for virus or VLP production (293T and 293F respectively) for 1h at room temperature with rotation. Next, IgGs purified from pre-adsorbed immunized animal sera were purified with prot A columns (GE Healthcare, Chicago, Il) and tested against a panel of 6 recombinant viruses from five different subtypes as described previously [39].

#### Statistics

For virion capture assays, statistical analysis was conducted by R using One-way NOVA followed by Newman Keuls post hoc test (***p<0.001 and *p<0.05). For the comparison of neutralization sensitivities to several bNAbs of viruses from neutralization HIV-1 database (http://hiv.lanl.gov/catnap) with any of the amino acid replacements incorporated by LR1-C1, statistical significance was evaluated with a Mann-Whitney U test. Simple comparisons were made with the use of a two-sided alpha level of 0.05. Endpoint ELISA titers were also evaluated with a Mann-Whitney U test and simple comparisons were also made with the use of a two-sided alpha level of 0.05.

## Results

### Generation and characterization of a randomly mutated envelope library (L)

**Fig 1:**
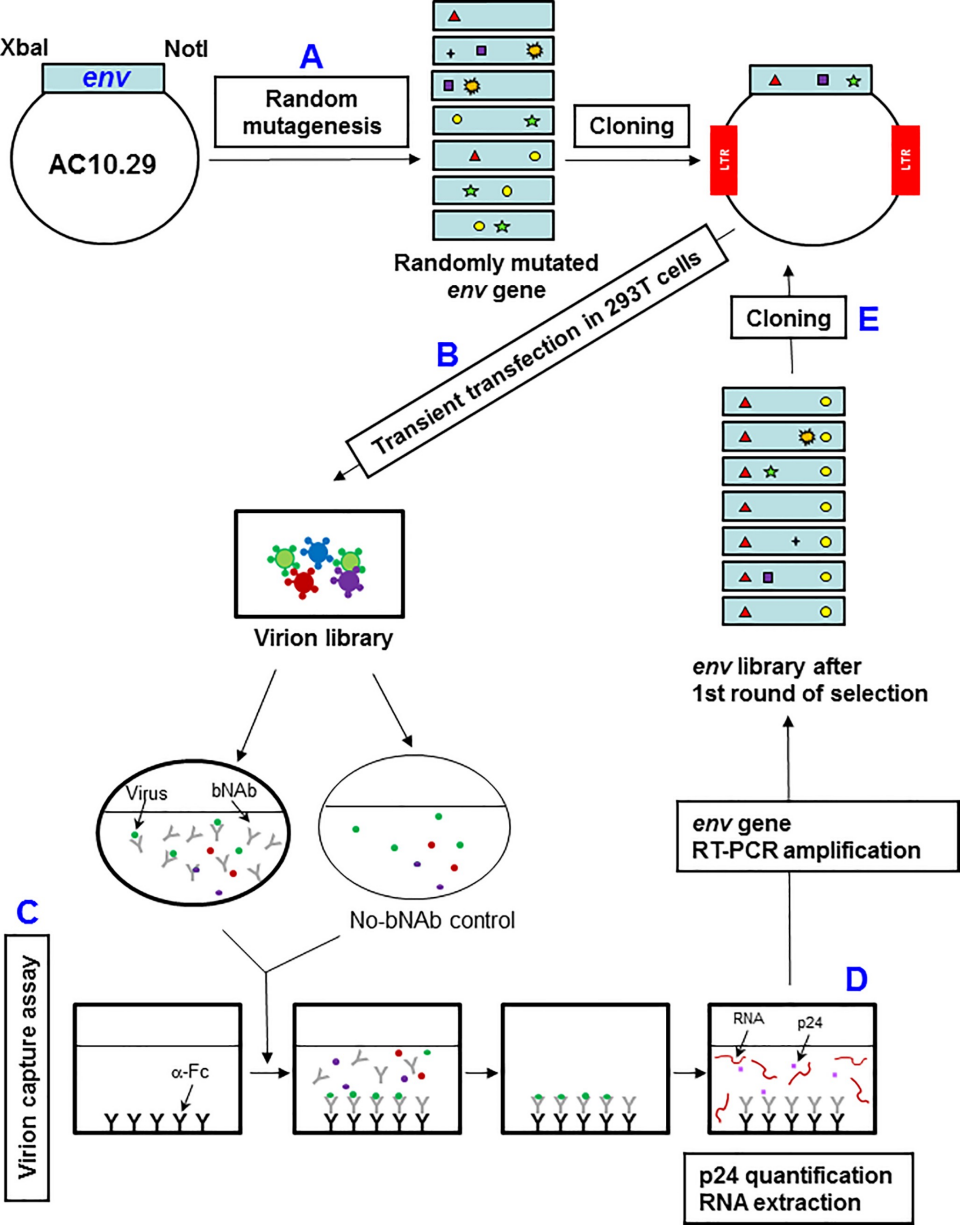
Rapid Immunogen Selection (R.I.S.) method. (A) Random mutations were introduced into HIV-1 *env* AC10_29 sequence by mutagenic PCR. (B) A library of chimeric HIV virions was generated by cloning randomly mutated envelopes into pNL4-3 using XbaI and NotI restriction sites and transient transfection into 293T cells. (C) Virion library was used in an enhanced virion capture assay modified from Leaman *et al* as indicated in the Materials and Methods section (41). (D) RNA from captured virions was extracted, retrotranscribed into DNA and amplified by nested RT-PCR. (E and B) A new library of chimeric virions was generated by transient transfection of the *env* amplified product out of captured virions and cloned into NL4-3 proviral DNA.

The objective of this study was the selection of Env variants with increased exposure of epitopes recognized by bNAb 4E10. This is an early antibody candidate that was available at the time of initiating selection experiments and humoral immunity to its targeted region had been associated with broad antiviral activity [40]. AC10_29 envelope from a clade B, tier 2 primary isolate was selected as the template for generating the randomly mutated library. AC10_29 was chosen for being a tier 2 primary isolate from a recently infected patient [41]. Tier 2 neutralization phenotype has been associated to closed Env trimer configuration that is believed to be more relevant for vaccination and is typical of most circulating strains [42]. AC10_29 overall neutralization profile against a group of bNAbs, including 4E10, is shown in S2 Table and our data are consistent with the data available at the CATNAP database (http://hiv.lanl.gov/catnap**)** [31]. Random mutagenesis was performed in the *env* gene by a PCR based method with conditions chosen in order to generate a mutation frequency of approximately 10^−3^ substitutions per nucleotide (Fig 1A). To monitor the mutations accumulated during random mutagenesis, the library (L) was cloned into pcDNA 3.1+ and the full-length *env* genomic nucleotide sequence from 15 clones was determined (Table 1). The frequencies of each mutation ranged between 22.3% for G→A and 1.5% for A→C. Non-synonymous mutations represented 47% of the total changes introduced. An insertion of one nucleotide and three single nucleotide deletions were also observed among the 15 clones (Table 1). In summary, 215 mutations were found (median; 14.33 and ranging from 3 to 21 mutations per *env* gene). Overall, mutation frequency in the library (L) was 5.5x10^-3^ substitutions per nucleotide calculated by comparison with the consensus sequence that matches AC10_29 and changes were randomly distributed throughout the *env* gene.

**Table 1:** Envelope gene mutations in virion libraries determined by standard and deep-sequencing before and after selection.

		Mutations													
		G→A	G→T	G→C	A→T	A→G	A→C	T→C	T→A	T→G	C→A	C→T	C→G	Del.^c^	Ins.^d^
Standard sequencing	L (%)^a^	22.3	7.9	4.2	13.0	7.9	1.5	8.8	13.0	5.6	6.5	5.1	2.3	1.5	0.4
	4E10sL (%)^a^	11.7	9.6	2.1	13.8	26.6	0.0	5.3	6.4	3.2	11.7	6.4	0.0	3.2	0.0
Deep-sequencing	L (%)^b^	19.4	6.6	1.4	12.1	6.4	1.6	11.6	11.7	0.0	3.7	21.1	3.4	0.9	0.0
	4E10sL (%)^b^	13.9	5.6	6.3	21.4	19.1	3.2	7.0	12.7	1.8	2.5	5.8	0.7	0.0	0.0

^a^ The *env* nucleotide sequence of 15 clones from the initial library (L) and 7 clones from the library after one round of R.I.S. selection with 4E10 monoclonal antibody (4E10sL) was determined as described in Materials and Methods section. The percentages are calculated over the total number of mutations.

^b^ More than 9,000 sequence reads were obtained for each of the 3 amplicons generated (S1 Fig) from both the initial library (L) and the library after one round of R.I.S. selection with 4E10 monoclonal antibody as described in Materials and Methods section. The percentages are calculated over the total number of mutations.

^c^ Nucleotide deletions

^d^ Nucleotide insertions.

**Fig 2:**
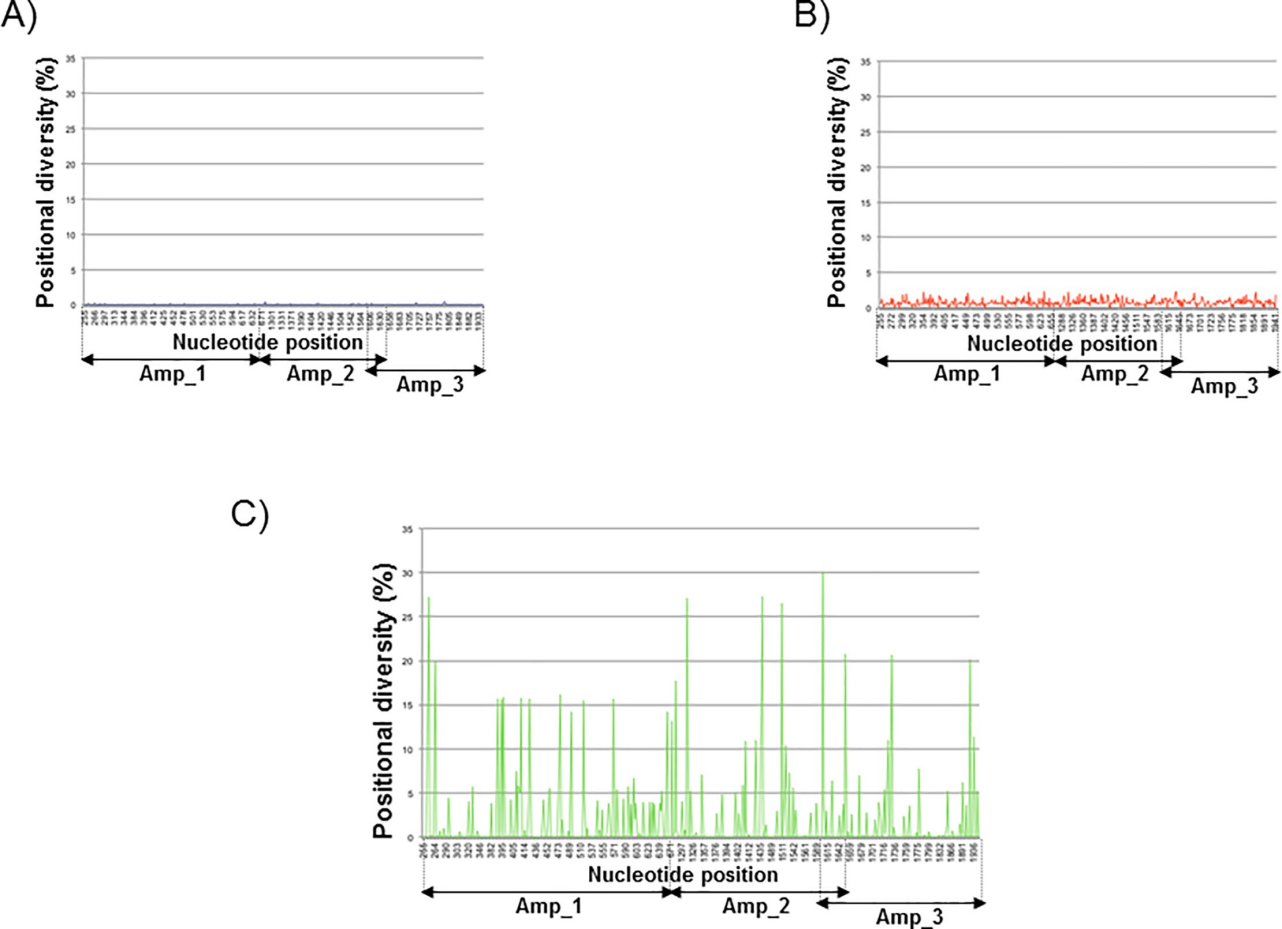
Positional diversity detected by deep-sequencing in parental virus AC10_29 (A) and the mutant library before [L; (B)] and after selection by the R.I.S. method [4E10sL; (C)]. (A) Amplicon sequencing of the original primary isolate before random mutagenenesis of the *env* gene revealed a homogeneous population. (B) The library generated by random mutagenesis had a wide variability of mutations across the amplicons sequenced. (C) Some mutations are fixed after selection with 4E10 by the R.I.S. method. Positional diversity is calculated as the frequency of all nucleotides that differ from the parental strain (AC10_29) at each evaluated position. Insertions and deletions are taken into account. Genomic regions corresponding to each amplicon are indicated.

Deep-sequencing analysis was used to characterize the library (L) in more detail, as previously described [43]. More than 9,000 454-sequence reads were obtained for each of the 3 amplicons generated from the parental clone and the library (S1 Fig). Considering the 454 sequencing error profile, diversity analysis was performed by counting non-reference nucleobases per nucleotide position on a reference-guided pair wise alignment. Positional diversity, defined as the frequency of all non-reference nucleobases at each position, for the parental clone was invariably below 0.3% except in two neighboring positions located in a homopolymeric region, which showed values around 0.6% (Fig 2A). Positional diversity analysis of the library (L) confirmed a random distribution of mutations in the dataset with no positional diversity higher than 3% (Fig 2B). The frequencies of each type of mutations observed by deep-sequencing were very similar to the frequencies obtained by standard sequencing (Table 1A and 1B). The only significant differences between standard and deep-sequencing analysis were the frequencies of T →G mutation (5.6% vs. 0.0%) and C→T (5.1% vs. 21.1%) (Table 1A and 1B). To determine whether the differences observed in both analyses could be attributed to the different regions sequenced by both methods (the full-length *env* gene for the standard sequencing and the regions corresponding to the three amplicons for deep-sequencing; S1 Fig), we compared the sequences only in the regions corresponding to the indicated deep-sequencing amplicons and similar results were observed (S3 Table). For this reason we believe that the observed differences could be attributed to cloning efficiency or to the limited number of clones analyzed by standard sequencing. In future studies we will increase the size of the sequenced library to address this issue.

### Mutation accumulation as result of selection of HIV-1 envelope mutants using 4E10 as bait with the Rapid Immunogen Selection method (R.I.S.)

To determine which mutations accumulated after 4E10 selection out of the randomly mutated *env* library (L), the 4E10 selected library (4E10sL) was cloned into pcDNA3.1+ and the full-length *env* genomic nucleotide sequence from 7 clones was determined. Mutation frequencies ranged between 26.6% for A→G and 0.0% for A→C and C→G; Non-synonymous mutations represented 90.4% of the total, in contrast to what was observed in the initial library (47%), which suggests a strong selection force by 4E10 selection. Three single nucleotide deletions were also observed (Table 1). In summary, 79 mutations were found (median: 11.29 and ranging from 8 to 22 mutations per *env* gene analyzed). Overall, mutation frequency in the library after 4E10 selection (4E10sL) was 4.3 x10^-3^ substitutions per nucleotide calculated by comparison with the corresponding parental sequence (AC10_29). Standard sequences have been deposited in GeneBank (accession numbers MK034104 to MK034126)

Next, a deeper analysis of the selected library (4E10sL) was carried out by deep-sequencing. 4E10 antibody-selected *env* sequences showed a significant increase of specific mutations compared to the initial clone that was used as template (Fig 2C). Deep sequencing experimental design was adapted to preliminary standard sequencing that revealed major differences in the deep-sequenced regions. Deep Sequencing rawdata have been deposited in CBI/SRA repository with accession PRJNA495005. Most of the amino acid replacements observed in the library after selection (4E10sL) with frequencies above 5% are apparently well tolerated by the envelope protein, as indicated by the acceptability value (Table 2). The degree of acceptability of the amino acid substitution follows the criteria described in Feng *et al*. where interchanges are more likely to happen between amino acids that have similar chemical structures [44]. The acceptability scale ranges from 0 to 6, with the latter value representing replacement by the same amino acid. Only 3 out of the 37 amino acid replacements observed with a frequency above 5% (D138Y, R503W and K574M) have an acceptability value below 3, supporting the notion of poorly accepted mutations. It is important to note that one third of the mutations enriched to above 5% frequency after selection were located in the gp120 V1 and V2 variable loops (Table 2).

**Table 2:** Amino acid replacements in AC10_29 parental sequence with frequencies above 5% observed in the envelope library after 4E10 R.I.S. selection (4E10sL).

Env amino acid change^a^^,^^b^	Frequency in 4E10sL (%)^c^	Acceptability^d^	Location^e^	Frequency in HIV-1 Database^f^ (LANL HIV-1 ENV AMINO ACID WEB; n = 6223 amino acid sequences)
E87G	17.8	4	C1	E:54.44%;G:15.15%;other;30,41%
N88D^g^	27.8	5	C1	N:99.21%;D:0.28%;other:0.79%
V89L	20.6	5	C1	V:98.18%;L:0.24%;other:1.58%
C131Y	16.3	3	V1	C:99.65%;Y:0.03%;other:0.32%
T132N	16.2	4	V1	T:61.10%;N:3.68%;other:35.22%
N137D^g^	7.8	5	V1	T:33.49%;N:31.98%;D:3.21%;other:31.32%
D138G	16.5	4	V1	N:27.27%;G:5.08%;D:3.82%;other:63.83%
D138Y	6.1	2	V1	N:27.27%;D:3.82%;Y:1.47%;other:67.44%
T141S	16.4	5	V1	N:27.66%;T:25.50%;S:13.66%;other:33.18%
F159Y	5.5	5	V2	F:96.88%;Y:2.79%;other:0.33%
N160Y^g^	17.0	3	V2	N:92.96%;Y:0.91%;other:6.13%
M165L	14.9	5	V2	I:46.42%;L:33.01%;M:4.19%;other:16.38%
Q172H	16.2	4	V2	E:42.84%;Q:3.41%;H:0.06%;other:53.69%
N187D^g^	16.3	5	V2	N:44.42%;D:16.46%;other:39.12%
S188aR	5.6	3	V2	Insertion amino acid R; not applicable
S195C	6.9	4	V2	N:67.72%;S:25.17%;C:0.04%;other:7.07%
I213F	5.4	4	C2	I:99.11%;F:0.09%;other:0.8%
Y217F	14.6	5	C2	Y:96.05%;F:3.05%;other:0.90%
A219V	13.5	5	C2	A:77.03;T:22.35%;V:0.36%;other:0.26%
A433T	6.2	5	C4	A:98.30%;T:0.17%;other:1.53%
S440G	10.8	5	C4	A:28.06%;S:14.41%;G:0.41%;other:57.12%
N462H^g^	8.1	4	V5	N:43.55%;S:12.35%;H:0.20%:other:43.90%
Q464E	8.4	4	V5	T:27.56%;E:11.0;Q:3.04%;other:58.40%
E466V	18.2	4	V5	E:96.38%;V:0.30%;other:3.32%
G472E	8.9	4	C5 (CD4 contact)	G:99.80%;E:0.04%;other:0.16%
M475I	13.6	4	C5 (CD4 contact)	M:90.87%;I:8.45%;other:0.67%
R500T	11.5	3	C5	K:49.77%;R:23.93%;T:3.65%;other:22.65%
R503W	15.4	2	C5	R:99.61%;other:0.39%; NO Ws
K510N	8.4	4	C5	K:97.80%;other:2.20%; NO Ns
S534T	26.2	5	gp41(HR1)	S:96.43%;T:0.032%;other:3.53%
V539I	7.1	5	gp41(HR1)	V:95.02%;I:0.04%;other:4.94%
L555P	7.9	3	gp41(HR1)	L:99.12%;other:0.88; NO Ps
T569I	5.4	3	gp41(HR1)	T:98.47%;other:1.53%; NO Is
K574M	23.9	2	gp41(HR1)	K:98.81%;other:1.19%; NO Ms
Q590P	8.8	3	gp41(HR1)	Q:99.58%;P:0.032%;other:0.38%
S618T	5.7	5	gp41(HR2)	S:76.31%;T:16.79%;other:6.90%
Y643N	12.1	3	gp41(HR2)	Y:98.15%;other:1.85%; NO Ns

^a^Amino acids are numbered according to the numbering for isolate HXB2.

^b^Replacements corresponding to LR1-C1 clone are shaded.

^c^Frequency of clones observed with each mutation out of the 9,000 sequence reads obtained for each of the 3 amplicons amplified from the library obtained after R.I.S. selection.

^d^The degree of acceptability of the amino acid substitution is given according to reference (42); the acceptability scale is from 0 to 6 with the latter value representing replacement by the same amino acid.

^e^Location of amino acid replacements is based on Los Alamos Database (www.hiv.lanl.gov)

^f^Amino acid alignments representing the fullest spectrum of sequences in Los Alamos Database (www.hiv.lanl.gov)

^g^ Potential N-linked glycosylation sites.

To create virions carrying selected *env* sequences after 4E10 selection, the library 4E10sL was subsequently cloned into the pNL4-3 backbone and sequenced. Sequence analysis of the resulting clones revealed that one of the clones, LR1-C1, incorporated a total of 10 amino acid replacements which were present in more than 10% (none of the amino acid replacements were present in a frequency below 10%) of sequences isolated after 4E10 selection (Table 2; LR1-C1 mutations are indicated). It is noteworthy that most amino acid replacements incorporated by LR1-C1 are rare according to their frequency in the HIV database (Table 2). 5 of these amino replacements (50%) were located in the gp120 V1/V2 loop. Mutation C131Y is especially relevant because this substitution eliminates the native disulfide bond between C131 and C157, thereby disrupting the architecture of the V1/V2 loop. Importantly, among the mutations incorporated in LR1-C1, there is a loss of 4 potential N-linked glycosylation sites: N88D, N137D, N160Y and N187D (Asn-X-Ser/Thr, where X can be any amino acid).

### 4E10 selected mutations incorporated by LR1-C1 increased virion affinity for 4E10, 2F5 and 447-52D monoclonal antibodies

**Fig 3:**
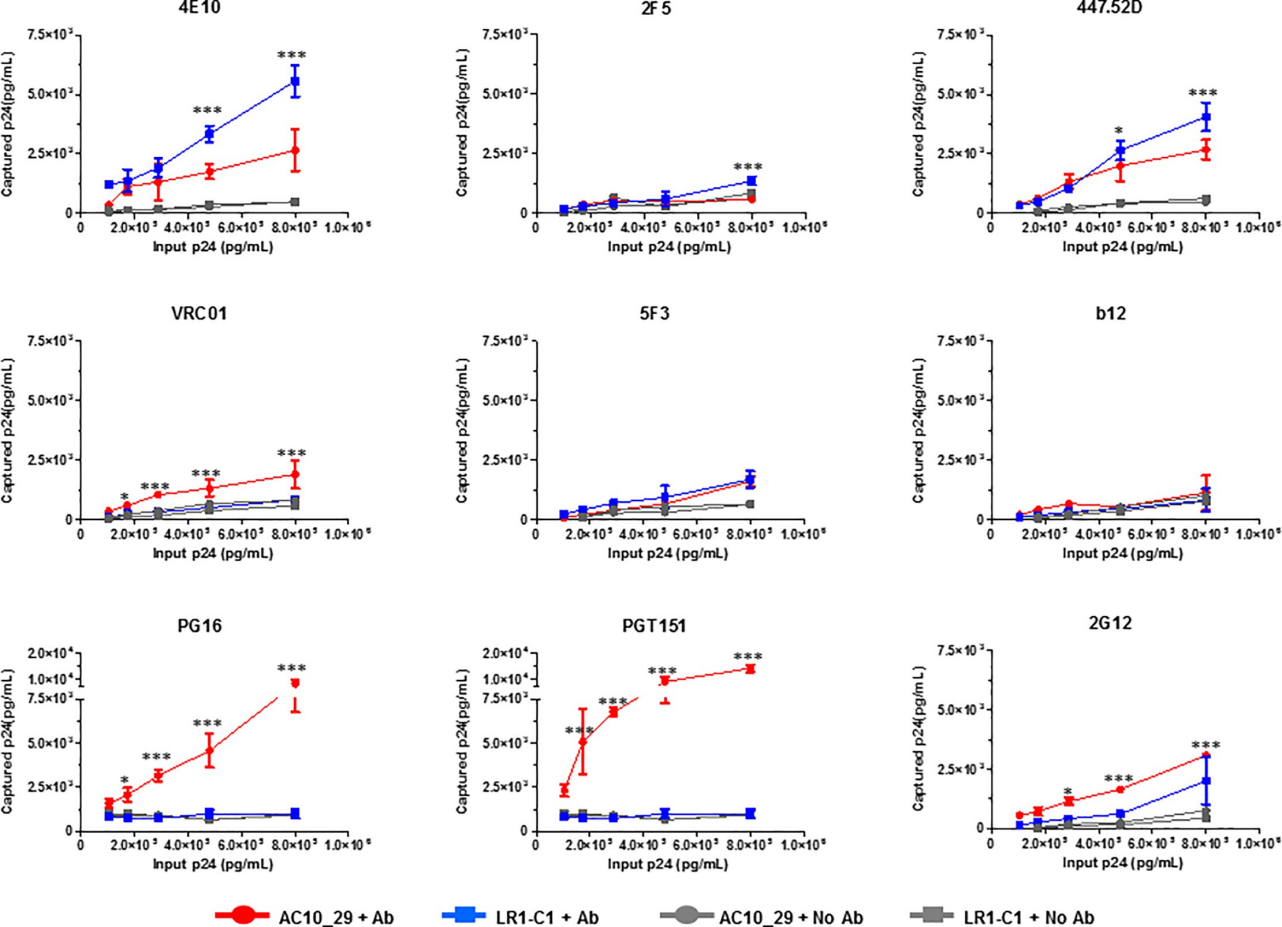
Effect of LR1-C1 mutations on affinity for selected bNAbs. Virus stocks were obtained from transfected 293T cells. Increase in Ab binding was determined by comparison of the virus captured by different monoclonal antibodies and quantified by p24 when similar amounts of virus (AC10_29 in red and filled circles and LR1-C1 in blue and filled squares) were used as input in a Virion Capture Assay (VCA) with the Abs 4E10 (MPER), 2F5 (MPER), 447-52D (V3), VRC01 (CD4bs), 2G12 (N332 V3 glycan patch), b12 (CD4bs), PG16 (quaternary; V1-V1 glycan apex), PGT151 (quaternary; gp120-gp41 integrase) and 5F3 (gp41). Similar amounts of virus suspension with no mAb were used as controls for both viruses (AC10_29 in grey and filled circles and LR1-C1 in grey and filled squares). Statistical analysis was conducted by R using One-way NOVA followed by Newman Keuls post hoc test at each input concentration (***p<0.001 and *p<0.05). Data are represented as mean ± SDEV.

The affinities of LR1-C1 variant for selected monoclonal neutralizing antibodies were compared to the parental virus (AC10_29) using a virion capture assay. The assay was performed in triplicate for each antibody at each virion concentration (Fig 3). The data showed an increased affinity of LR1-C1 virions for 4E10, 2F5 and 447-52D antibodies compared to the parental virus. A statistically significant increase in affinity was also observed for antibody 2F5 only at the highest input concentration (*p<0*.*001*). Noteworthy, a statistically significant affinity increase was also observed for antibody 4E10 at two input concentrations *(p<0*.*001)* confirming the efficacy and specificity of the selection process. As confirmation of this result we have observed that LR1-C1 also has increased affinity for antibody 10E8 that is specific for the same epitope (S3 Fig). Additionally, a significant increase was observed for 447-52D at two input concentrations (p<0.05 and <0.001 respectively). The increased affinity of 4E10 selected virions for the 2F5 antibody is consistent with the close proximity in which the two antibody epitopes are located inside the gp41 membrane proximal domain. These affinity increases are also in line with several reports that correlated the loss of glycosylation sites and the disruption of the V1-V2 loop architecture with an increased sensitivity to antibody mediated neutralization [38,45–52]. The increase in the affinity for 447-52D, whose epitope is located in the V3 loop, is also consistent with previous reports that showed the masking of V3 by the V1-V2 loop [53–57]. However, the increased binding by 447-52D suggests that LR1-C1 Env may have changed to an open tier 1-like conformation [58]. The affinities for VRC01 and 2G12, in contrast, decreased compared to AC10_29 and no binding to PG16 and PGT151 was observed for the LR1-C1 variant. The lack of binding to PG16 and PGT151 could be attributed to mutations at positions 160 and 503 respectively that have been previously associated to this effect [59,60]. The decreased exposure of additional epitopes, such as VRC01, 2G12, PG16 and PGT151, that could also be relevant for the induction of broadly neutralizing antibodies could be an undesired side effect associated with the selection process. The low affinity of LR1-C1 to quaternary-structure dependent antibodies, such as PG16 and PGT151, suggests that LR1-C1 envelope may be inefficient forming trimers. No differences in the affinity for 5F3 antibody (non-neutralizing; gp41 C34 helix) were observed.

### Amino acids incorporated by LR1-C1 are associated with increased sensitivity to 4E10 and 2F5 antibodies in HIV-1 isolates described in the Los Alamos database

**Fig 4:**
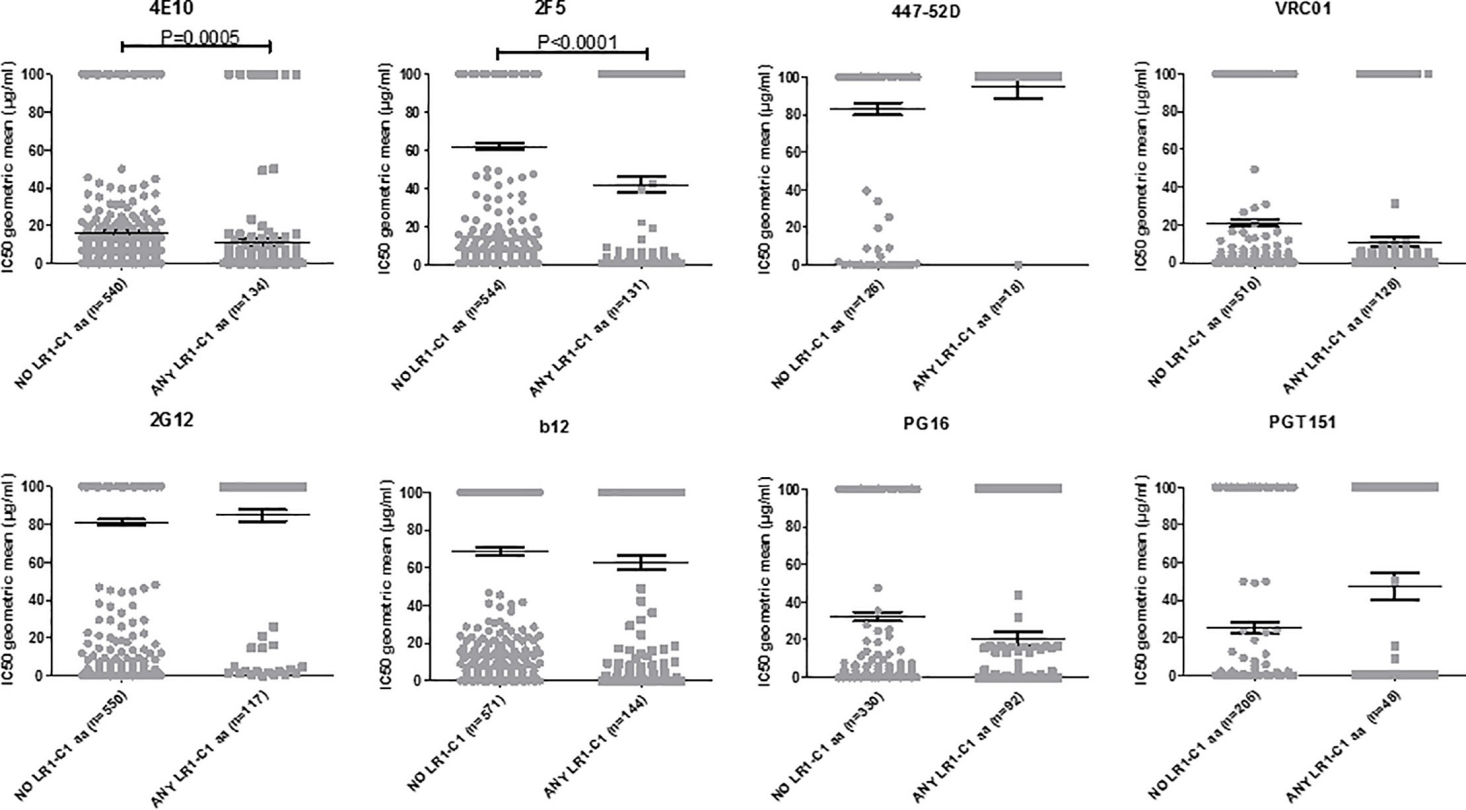
Neutralization sensitivity to bNAbs of viral isolates with 4E10-selected aminoacids. Graphical plot showing the comparison of neutralization sensitivities to several bNAbs of viruses from neutralization HIV-1 database (http://hiv.lanl.gov/catnap) with any of the aminoacid replacements incorporated by LR1-C1 that are indicated in Table 2 (closed grey squares) and with none of the aminoacids incorporated by LR1-C1 (closed grey circles). HIV-1 isolates with Y131, W503 and N643 aminoacids were not included in the analysis because there was no neutralization data or no isolate with this mutation in the database. Mann-Whitney U test was used for comparisons of continuous variables between groups. Simple comparisons were made with the use of a two-sided alpha level of 0.05.

First of all we wanted to determine the association of 4E10 selected amino acids with increased neutralization sensitivities to selected monoclonal antibodies by TZM-bl assays. Unfortunately, this analysis could not be done because LR1-C1 was not infectious in TZM-bl cells (S2 Fig). As an alternative approach, we compared the corresponding IC_50_s between viruses that had one or more of the amino acid replacements incorporated by LR1-C1 (Table 2) and viruses that did not contain any of those amino acids in HIV-1 isolates from Los Alamos database. Our analyses showed that the IC_50_ titer of 4E10 and 2F5 antibodies were significantly higher among the virus group with one or more LR1-C1 amino acids compared to the group with no LR1-C1 amino acids (p<0.005 and p<0.0001 respectively; Fig 4). However, IC_50_ titers of 447-52D, VRC01, 2G12, b12, PG16 and PGT151 did not significantly differ between the virus groups with one or more LR1C1 amino acids or no LR1-C1 amino acids (Fig 4). HIV-1 isolates with Y131, W503 and N643 amino acids were not included in the analysis because there were no isolates with these amino acids or no neutralization data in the database.

### Effects of mutations incorporated after 4E10 selection on envelope incorporation in virions

**Fig 5:**
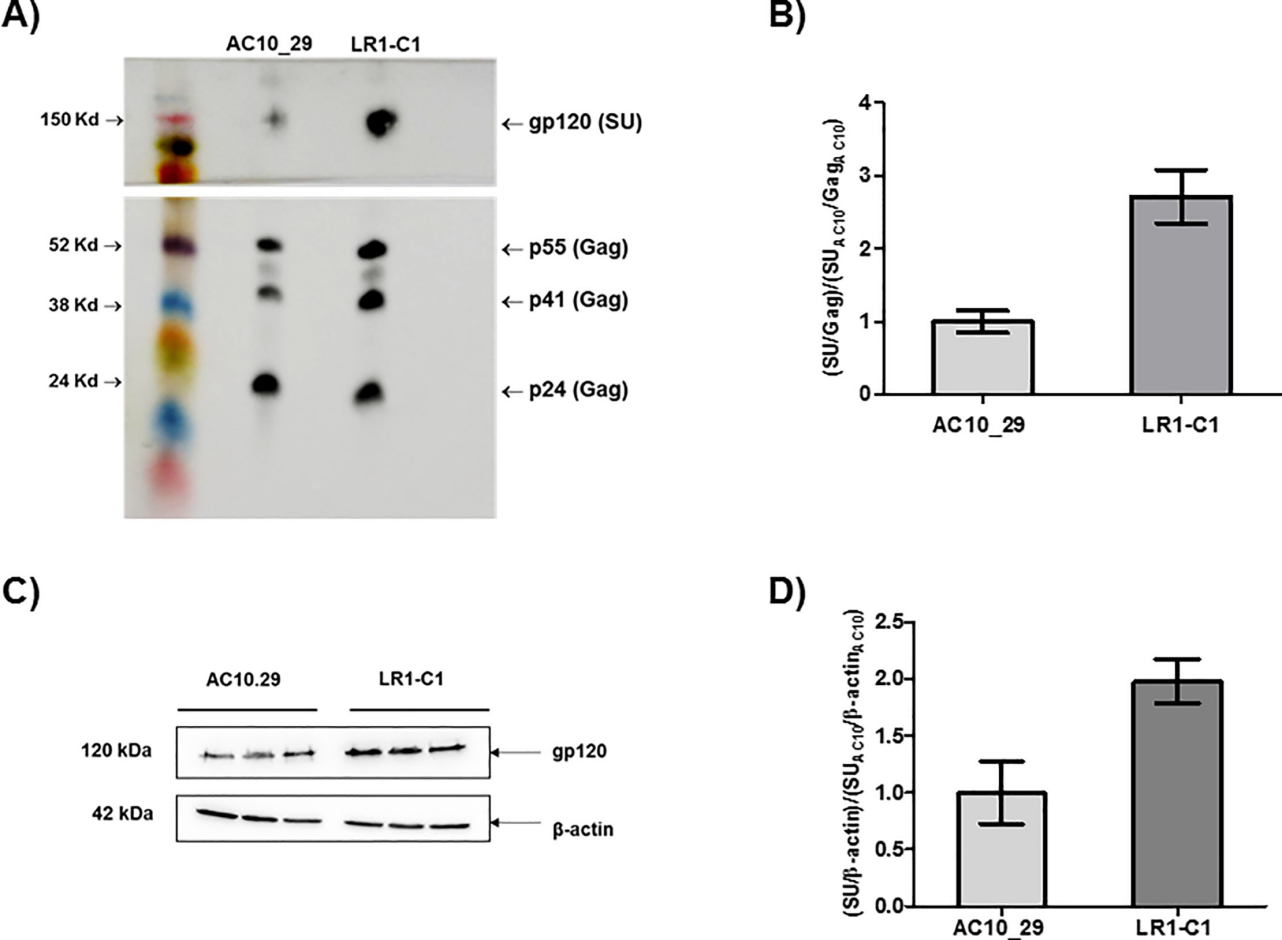
Effect of different mutations in Env on incorporation into virions. Viruses were produced by transfection into 293T cells, virions were chemically inactivated with AT-2 and pelleted from the clarified supernatants. (A) SU (gp120) and Gag (p55, p41 and p24) proteins were detected by Western blotting using 447-52D and anti-p24 monoclonal antibodies. (B) Ratios of SU to Gag (p55+p41+p24) were calculated by using phosphorimaging analysis. Virion producer cells were lysed as indicated in the Materials and Methods section. C) SU and β-actin proteins were detected by Western blotting using 447-42D and anti-β-actin monoclonal antibodies. D) Ratios of SU to β-actin were calculated by phosphorimagiing analysis. Data for LR1-C1 are presented relative to the ratio found for AC10 _29 virions.

Next, we assessed the extent to which mutations incorporated by LR1-C1 affected incorporation of envelope proteins into virions. To this end, infectious virus was produced in 293T cells, chemically inactivated with AT-2 and pelleted from clarified supernatant. Virion samples were then subjected to analysis by Western blot (Fig 5A). This analysis revealed a significant amount of virions with incomplete processing of Gag precursors evidenced by the presence of p55 and p41 Gag proteins. This effect could be attributed to the AT-2 treatment considering that inhibitors of HIV-1 zinc fingers prevent normal processing of Gag precursors [61]. In addition, we determined the Env to Gag (p55+p41+p24) ratio in virions carring the 4E10-selected envelope, LR1-C1, relative to the AC10_29 by densitometry (Fig 5B). This analysis revealed that LR1-C1 envelope was incorporated into virions at higher levels than AC10_29 (2.7-fold increase; Fig 5). Finally, Env expression in 293T virion producer cells was analyzed and showed a 2-fold increase in Env expression per cell that could be, at least in part, responsible for the greater Env incorporation in LR1-C1 virions (Fig 5C and 5D).

### 4E10-selected envelope variant (LR1-C1) increases the neutralization capacity of the humoral response induced in BALB/c mice

Changes in the humoral immune response following immunization with the AC10_29 wild type or the LR1-C1 variant were evaluated in mice vaccinated with AT-2 inactivated virions. AT-2 inactivation was chosen over heat-inactivation because this procedure better preserves Env three-dimensional structure [62]. In order to deplete the sera of vaccinated animals from the antibodies generated against cellular components present in virions (from 293 producer cells), sera from immunized mice were pre-adsorbed against 293T cells. In order to minimize the background, IgGs were purified from immunized mice and were tested against a VSV pseudotyped HIV-envelope deficient control. Purified IgG fractions were then tested for neutralizing activities against two different HIV-1 strains (NL4-3, tier 1 and AC10_29, tier 2). Assays were run in triplicate at a 0.2mg/ml IgG concentration and neutralization results are shown in Table 3. IgGs purified from mice immunized with LR1-C1 showed a moderately improved neutralization breadth compared to the AC10_29 immunized mice. Three out of the five sera were capable of neutralizing AC10_29 and one of them could also neutralize NL4-3. In contrast, only one mouse of the 5 animals immunized with AC10_29 neutralized AC10_29 virus with no neutralization of NL4-3 at all. IgG purified from immunized mice sera showed no neutralization of the VSV pseudotyped virus used as specificity control. Due to limited sample size, insufficient purified IgGs were obtained from immunized mice and complete neutralization curves could not be performed. Therefore, IC_50_ values could not be determined and further neutralization testing of against additional HIV isolates and epitope mapping could not be performed.

**Table 3:** Neutralization of AC10_29 and LR1-C1 immunized mice.

	AC10 immunized mice				LR1-C1 immunized mice				
	1	2	3	4	1	2	3	4	5
VSV pseudotyped	8.2±5.6	11.1±11.2	0.0±10.7	15.0±4.2	7.6±16.3	0.0±6.1	3.6±1.5	0.0±11.2	0.0±16.5
NL4-3	0.0±0.5	3.0±25.8	0.0±18.9	33.3±30.1	58.8±14.6	0.0±11.7	0.0±41.5	0.0±28.3	33.6±17.3
AC10	13.9±23.7	47.0±3.7	47.3±5.7	60.0±3.8	63.8±1.8	32.9±9.6	65.0±10.9	44.3±5.4	54.7±5.2

Numbers indicate percent neutralization ± standard deviation. A white box indicates <50% neutralization and a yellow box indicates >50% neutralization.

### 4E10-selected envelope variant (LR1-C1) increases immunogenicity of the 4E10-proximal region in rabbits

**Fig 6:**
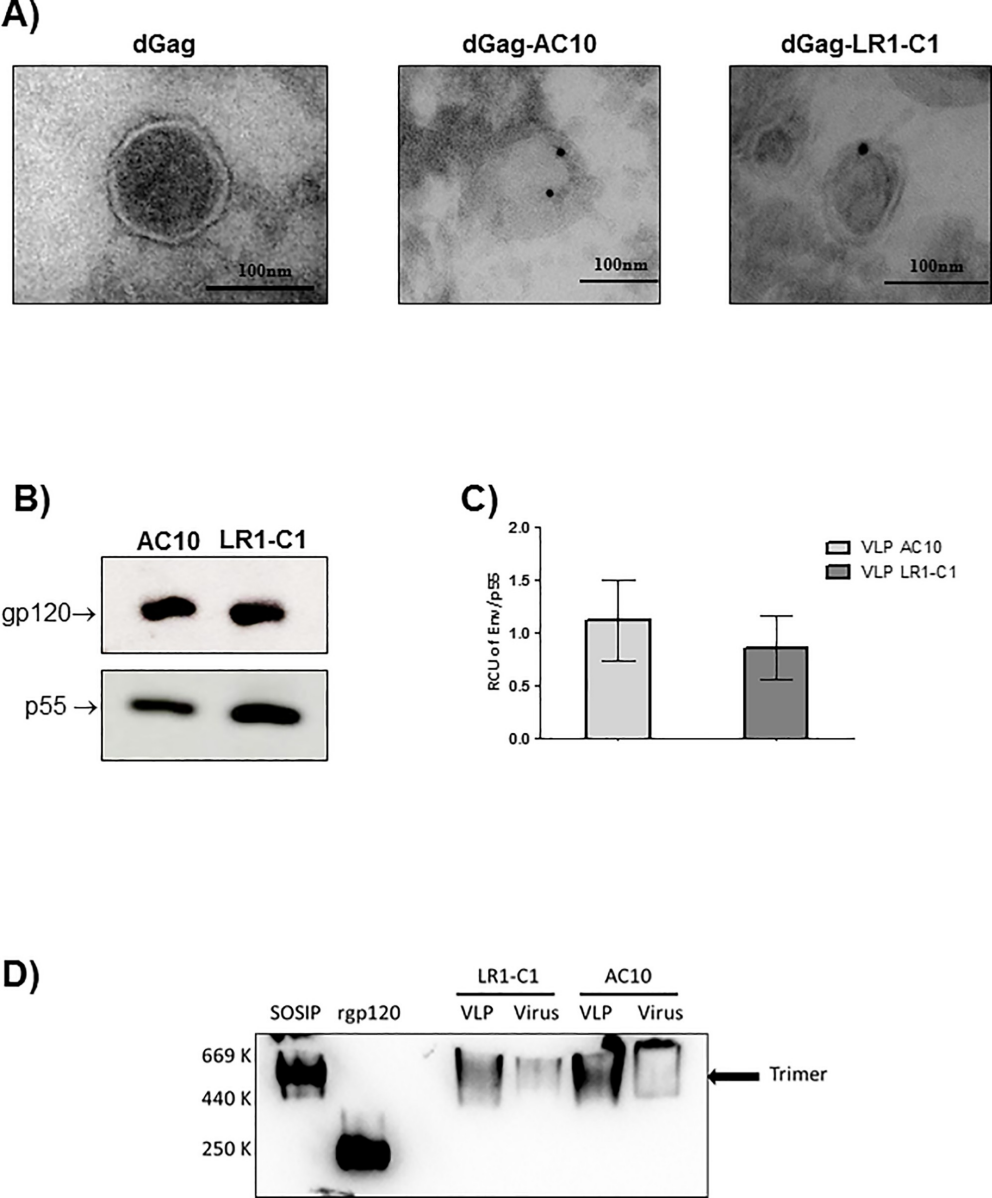
Characterization of the VLPs used for rabbit immunization. VLPs were produced by transfection into 293F cells and VLPs were pelleted from the clarified supernatants. (A) VLPs were analyzed by immunocrioelectromicroscopy labelling with an anti-gp120 antibody, 447-52D. (B) Envelope incorporations in AC10_29 and LR1-C1-VLPs were evaluated by the gp120 and p55 ratios by western blotting in SDS-PAGE gels using 447-52D and p24 (24–4) monoclonal antibodies. (C) Relative Env incorporation into VLPs was calculated by the ratios of gp120 to p55 using phosphorimaging analysis in triplicate. (D) The presence of trimers into AC10_29 and LR1-C1-VLPs and AC10_29 and LR1-C1-virions were analyzed by western blotting in BN-PAGE gels. The different forms of Env were labelled using a anti-gp120 and anti-gp41 cocktail (2G12, b12, 39F, 2F5, 4E10 and 447-52D). As controls, a 96ZM 651_HXB2_V3 SOSIP and a rgp120 were used.

To further evaluate the humoral immune response induced by the 4E10-selected envelope, rabbits were immunized with HIV-1-Gag virus like particles carrying AC10_29 and LR1-C1 envelope variants. VLPs were generated using the dGag sequence and each envelope sequence as indicated in the Materials and Methods section. Cryo-immuno Transmission Electron Microscopy (TEM) was conducted to analyze VLPs produced from 293F cells that had been transiently transfected with Gag and Env expression vectors as indicated in the Materials and Methods section. Immuno-electron cryo-microscopical analysis revealed that VLPs were spherical with electron density below the VLP membrane. In addition, VLPs resulted in particle sizes around 130nm in diameter and envelope incorporation was verified by 447-52D labelling (Fig 6A). The amount of labeling per VLP was scarce, as is expected of HIV envelope proteins with untruncated gp41 cytoplasmic tails [63]. Transfection conditions were adapted to ensure that both VLPs had similar amounts of envelope incorporated and was verified by western blotting (Fig 6B). The presence of either envelope trimers incorporated into VLPs and was confirmed by BN-page (Fig 6C) showing comparable levels of AC10_29 and LR1-C1 in these VLP preparations.

**Fig 7:**
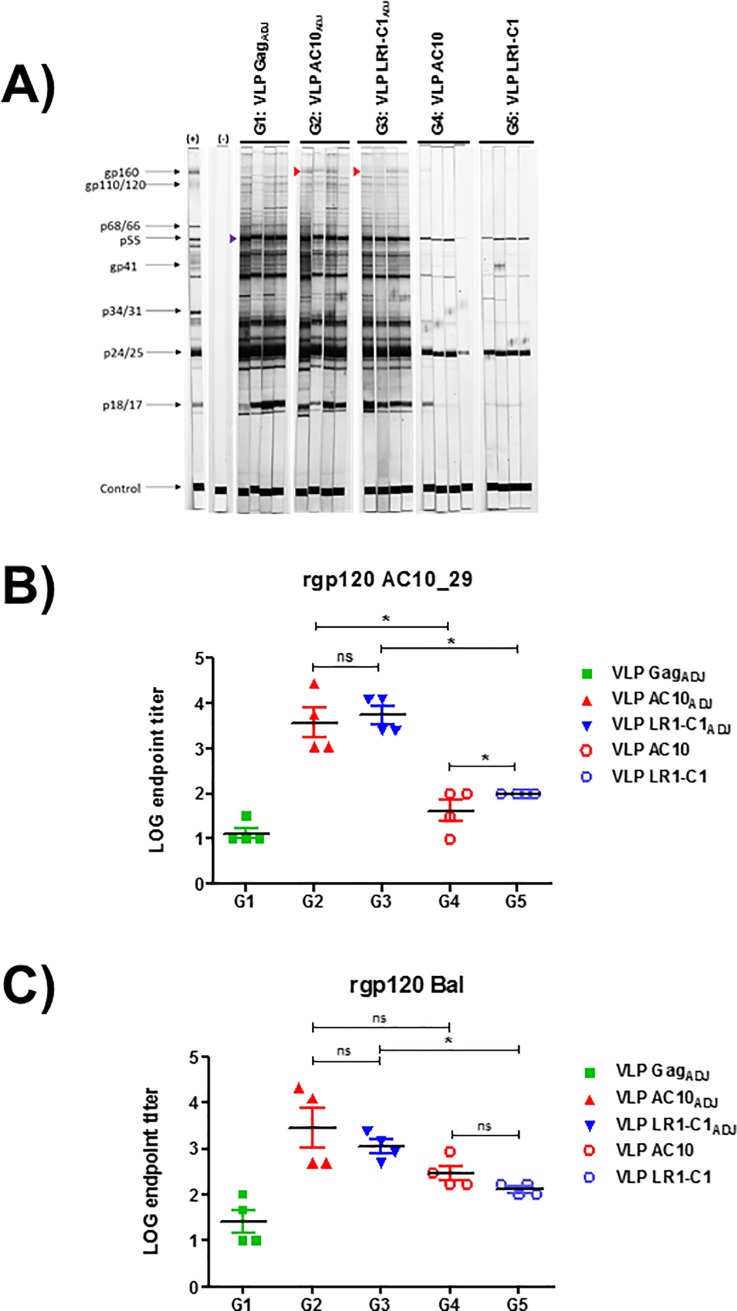
Characterization of the humoral responses induced in immunized rabbits. The humoral immune response of the immunized rabbits was characterized at week 15. (A) The presence of anti-HIV antibodies in rabbit sera were analyzed by western blot. Preimmune sera of rabbit 1640 was also included as an additional negative control. Endpoint ELISA titters to autologous (AC10.29; B) and heterologous (Bal; C) rgp120. Open symbols correspond to animals immunized with no adjuvant. The highest dilution which gave an OD_450_ 2.5 folds higher than pre-immune sera without dilution was designated as the antibody endpoint titter. Results showed data of three independent experiments and results were expressed as the mean ±SD (n = 4). Ns:non significant; *p<0.05 Mann-Whitney U test.

**Fig 8:**
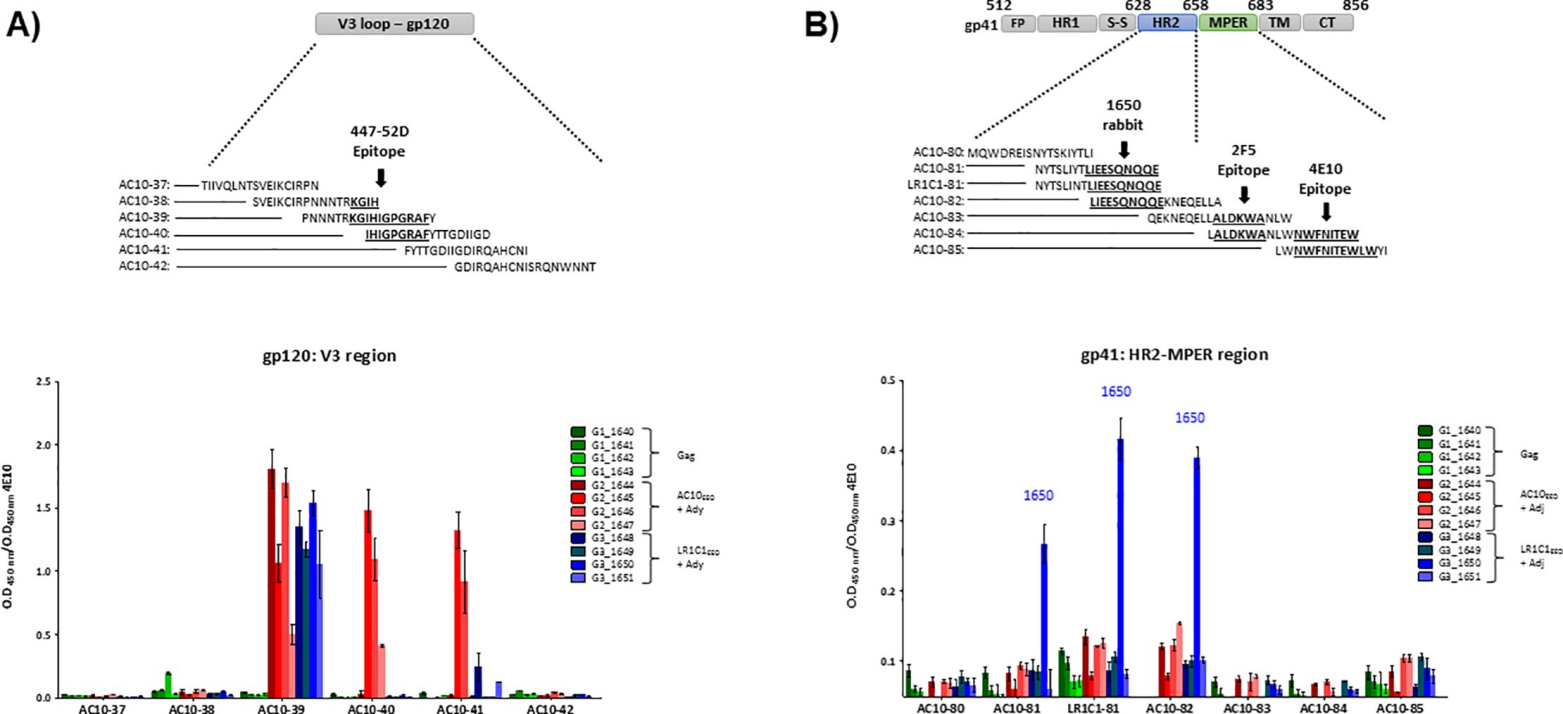
Characterization of the antibody responses against the V3-loop and the gp41 ectodomain regions. (A) Reactivity of sera from immunized rabbits to the V3 loop was analyzed by ELISA of overlapping 15-mer peptides corresponding to the V3 loop of the AC10_29 isolate. Peptide sequences are shown and the 447-52D epitope is indicated. (B) Reactivity of sera from immunized rabbits to the gp41 ectodomain was analyzed by ELISA of overlapping 15-mer peptides corresponding to the gp41 ectodomain of the AC10_29 isolate and one peptide with the LR1C1 sequence. Peptide sequences are shown and the 4E10 and 2F5 epitopes are also indicated.

Rabbits were immunized with VLPs carrying AC10_29 and LR1-C1 envelopes with and without adjuvant (G2: AC10_29 with adjuvant; G3: LR1-C1 with adjuvant; G4: AC10_29 with no adjuvant and G5: LR1-C1 with no adjuvant). Adjuvanted HIV-1-Gag VLPs with no envelope incorporated were used as controls (G1). All rabbits immunized with AC10_29 and LR1-C1 HIV-1-Gag-VLPs developed responses to HIV-1 Gag and Env HIV-1 proteins (Fig 7). Responses to monomeric rgp120 were more potent to both autologous and heterologous rgp120 when Adjuplex was used for immunization (G2/G3; Fig 7B and Fig 7C). In addition, the gp120 responses were slightly higher in the LR1-C1-immunized group compared to the AC10_29 immunized group only with autologous gp120 but these differences were statistically significant only in the groups immunized with no adjuvant. All 8 sera from G2 and G3 rabbits showed antibody responses to the gp120 V3 loop (Fig 8A) and one of the rabbits immunized with LR1-C1 HIV-1-Gag-VLPs (1650) developed an antibody response to a region in close proximity to the 4E10 epitope (18 amino acids upstream; Fig 8B, specifically against C-terminal heptad repeat (CHR) region of gp41 protein). No neutralizing activity against any of the viruses tested was observed in purified IgGs from the immunized rabbit sera.

## Discussion

The success of passive transfer studies with SHIV infection in macaques and HIV infection in small-animal models and in therapeutic setting in humans suggest that bNAbs have the potential to prevent viral infection [13,64]. Despite these achievements, the field continues to face significant hurdles for the development of an antibody-based vaccine. The approach of reverse vaccinology, by which knowledge of the molecular structure of antigens is built into unnatural immunogens to elicit neutralizing epitopes [65–67], may help overcome some of these road blocks. Following this approach, several envelope based immunogens have been designed and tested experimentally. These include trimeric soluble gp140 molecules containing the extracellular portion of gp41 coupled with gp120, which have in some cases proven to be immunogenic, but overall have not elicited strong broadly neutralizing responses.

Random mutagenesis has been widely used by researchers to identify beneficial mutations in the absence of structural information, or when such mutations are difficult to predict from protein structure [68]. In the present study we decided to use this strategy for improving the accessibility of the epitopes recognized by selected broadly neutralizing antibodies. This is based on the hypothesis that an envelope with increased affinity for a bNAb will bind more efficiently to the B-cells expressing IgGs with the corresponding specificities thereby improving the clonal expansion of the corresponding B cell clones. Therefore, the selected envelope would be more efficient as an immunogen capable of inducing antibodies against the corresponding envelope region than its wild-type version. In this scenario, long periods of somatic mutation would not be required to increase antibody-epitope affinity for bNAb induction. Furthermore, the identification of HIV-1 infected patients with broadly neutralizing activities within the first months of infection indicate that long periods of somatic mutation may not always be essential to induce bNAbs[10].

Here, we have used this approach and successfully selected an envelope variant sequence with increased affinity for the bNAb 4E10 after just one round of selection. The selected envelope (LR1-C1) has an increased affinity for the antibody used as bait (4E10). The amino acid replacements incorporated by this variant, the loss of N-linked glycosylation sites and change in the architecture of the V1/V2 loop, were all consistent with changes that have been previously associated with increased exposure of neutralizing epitopes and which has been linked to increased neutralization sensitivity and increased induction of neutralizing antibodies [45,46,48–51]. The increased affinity of several epitopes might also be explained by the selection of an envelope with an open conformation (tier 1-like). This hypothesis would be supported by the observation of an increased binding to 447-52D [58]. Additionally, the affinity increases in several epitopes observed could also be due to the 2.7 fold increase in envelope incorporation observed in LR1-C1. Nevertheless, we hypothesize that there is some specificity associated with the affinity increases detected taking into consideration that they do not affect all the epitopes in the same way. In fact, LR1-C1 has a decreased affinity for 2G12 and VRC01. Moreover, we found an association between the 4E10-selected amino acids incorporated in the LR1-C1 variant and increased sensitivity to 4E10 and 2F5 when more than 650 HIV-1 isolates from the Los Alamos database were analysed (674 for 4E10 and 675 for 2F5). Furthermore, in the RV144 vaccine trial efficacy has been associated to non-neutralizing antibodies targeting positions 169 and 181 within the V2 loop that are in close proximity to mutations N160Y and N187D incorporated in our selected envelope [69]. The observation that the mutations we have identified in our study and mutations associated to vaccine efficacy in RV144 vaccine trial are proximal may indicate that, this V2 loop region could be a site of vulnerability relevant for induction of antibodies with both neutralizing and antibody-dependent cell-mediated cytotoxic activities. Vaccination strategies based on these immunogens should bear in mind the possible decreased exposure of additional relevant epitopes that could be circumvented by combinations of envelopes with increased exposure of different epitopes.

One aspect that could have limited the success of this method would be associated to a loss of high-affinity clones as a result of the multiple cloning steps required by the selection strategy. An additional limitation would be the low potency of the antibody chosen for selection. Despite all these limitations, our data are consistent and the relevance of improving the exposure of the 4E10 region has been confirmed later thanks to the identification of antibody 10E8, that recognizes the same epitope with greater amplitude and potency [65]. In fact, in our study, the 4E10 selected envelope also has an increased affinity for 10E8 antibody (S3 Fig).

Our data are in line with the general belief that extensive glycosylation of the external surface component of Env gp120 contribute importantly to its poor immunogenicity. The gp120 surface glycoproteins of HIV and simian immunodeficiency virus (SIV) each contain approximately 24 sites for N-linked carbohydrate attachment (Asn-X-Ser/Thr). The dispensability of some N-linked glycans for viral replication and the greater sensitivity of some glycan-deficient mutants to antibody-mediated neutralization suggest that these glycans may serve in part as barriers to shield the virus for effective antibody recognition [38,45,46,48–52,70]. Additionally, the observation that amino acid replacements associated to increased exposure of 4E10-epitope are associated, not only to an increased sensitivity to the 4E10 antibody, but also to 2F5 are consistent with the proximity of both epitopes in the viral envelope protein. On the other hand, the role of variable gp120 loops, particularly the first and second variable loops (V1/V2 region) and the third variable loop (V3) in immunogenicity and antibody evasion have been widely characterized by different groups suggesting that these domains contain epitopes that are targets for neutralizing antibodies and shield different neutralizing epitopes[71–74]. Inherent neutralization resistance of several HIV strains is to a great extent mediated by gp120 V1/V2 domain structure rather than by sequence variations at the target sites. Experimental and cryo-EM structural evidence supported the hypothesis that V1/V2 in one gp120 monomer masks V3 on the same monomer (*cis*) [75,76].

In this study, the antibody response induced in the mice model by the 4E10-selected envelope variant was enhanced compared to the response induced by the virus carrying the original envelope sequence. Unfortunately, the breadth of the neutralizing response, as well as the mapping of the humoral response induced, could not be assessed due to limitations in sample availability. In order to bypass this limitation, we decided to evaluate the immunogenicity of the 4E10-selected variant in a larger animal model that yield sufficiently large blood samples. However, in that model, animals did not mount a detectable level of neutralization activity. Furthermore, one out of the four rabbits immunized with the 4E10-selected variant redirected antibody response toward a region proximal to the epitope recognized by the antibody used for selection (Fig 8). Moreover, the region to which the antibody response has been redirected is recognized by 2F5-like antibodies [77]. Possible factors that could explain the absence of neutralizing response include the conformation or the amount of the envelope spikes incorporated into the VLPs and the immunization strategy used, which may not be the most effective approach for the induction of broadly neutralizing antibody responses. In particular, we have generated an envelope variant with potential to better present a specific epitope and, therefore, a potential greater capacity to induce responses directed to the corresponding epitope. For this reason, the immunization strategy chosen included short periods of antigen exposure. However, a longer affinity maturation process may be required with the current prototype [78,79]. Additionally, there are evidences indicating that gp41 bnAbs are controlled by immune tolerance and require vaccination with regimens designed to transiently dampen immune tolerance [80]. Introduction of mutations that stabilize trimers, enzymatic digestion to remove spikes with aberrant conformation or serial booster vaccination may further help to overcome these limitations [81]. Another way to improve the method would be by selection with quaternary-structure dependent antibodies that would increase the likelihood of selecting variants with intact trimers that would be better inducer of neutralizing antibodies [82,83].

The results of this study are a proof of concept indicating that the method described here allows us to select, without using any previous structural information, variants with greater exposure of certain epitopes of interest to induce more effective humoral immunity. Moreover, the results shown in here can serve as a starting point for the development of new immunogens through selection with new and possibly multiple different bNAbs, the incorporation of successive rounds of selection, or the generation of new libraries from different HIV subtypes. These steps could aid the development of an HIV vaccine through the selection of mutant envelopes and their delivery by incorporation in appropriate vectors. In addition, the present approach could be taken one step further adapting it to the development of immunogens for other viral infections.

## Supporting information

S1 Fig Amplicon design for deep-sequencing of HIV-1 *env*. Deep-sequencing of the different Env variants was achieved by sequencing of 3 PCR amplicons (Amp_1, Amp_2 and Amp_3) covering regions of interest.
(TIF)

S2 Fig Effect of LR1-C1 mutations on viral infectivity. Virus stocks were obtained from the transfection of 293T cells. Stocks were normalized to the amount of p24 and used to infect TZM-bl cells. Luciferase activity (L.U.) was measured as described previously [39].
(PPTX)

S3 Fig Effect of LR1-C1 mutations on affinity for antibody 10E8. Virus stocks were obtained from transfected 293T cells. Increase in 10E8 binding was determined by comparison of the virus captured by the antibody and quantified by p24 when similar amounts of virus (AC10_29 in red and filled circles and LR1-C1 in blue and filled squares) were used as input in a Virion Capture Assay (VCA). Similar amounts of virus suspension with no mAb were used as controls for both viruses (AC10_29 in grey and filled circles and LR1-C1 in grey and filled squares). Statistical analysis was conducted by R using One-way NOVA followed by Newman Keuls post hoc test at each input concentration (***p<0.001 and *p<0.05). Data are represented as mean ± SDEV.
(TIF)

S4 Fig Prime-boost protocols for mice immunization. Balb/c mice were inoculated at weeks 0 and 2 and bled at week 4. Five animals per group were used. Five prime-boost immunization protocols were followed. Both groups were inoculated with 100μl of AT-2 inactivated virus at weeks 0 and 2 (2.5μg/ml p24 quantified as explained in the Materials and Methods section) and 100μl of complete Freund adjuvant subcutaneously). Group 1 was inoculated with AC10 AT-2 inactivated virions and group 2 was inoculated with LR1-C1 AT-2 inactivated virions. At week 4, animals were bled by cardiac puncture with anesthesia from both groups. These experiments were performed at BSL3 CReSA Biocontainment Facility with the collaboration of CReSA staff (Barcelona, Spain).
(PPTX)

S5 Fig Prime-boost protocols for immunization of rabbits. Animals were inoculated at weeks 0, 3, 6, 9, 12 and bled at week 15. Four animals per group were used. Five prime-boost immunization protocols were followed. In group 1 (animals from 1640 to 1643) plasmid pcDNA-Gag+pcDNA (250μg/animal each) were boosted twice with dGag VLPs using adjuplex as adjuvant. In group 2 (animals from 1644 to 1647) plasmid pcDNA-Gag+pcDNA-AC10_EEO_ (250μg/boost/animal each) and animals were boosted twice with dGag-AC10 VLPs (20μg of p24/boost/animal) using adjuplex as adjuvant. In group 3 (animals from 1648 to 1651) plasmid pcDNA-Gag+pcDNA-LR1C1_EEO_ (250μg/animal each) and animals were boosted twice with dGag LR1C1 VLPs using adjuplex as adjuvant. In group 4 (animals from 1652 to 1655) plasmid pcDNA-Gag+pcDNA-AC10_EEO_ (250μg/animal each) and animals were boosted twice with dGag-AC10 VLPs with no adjuvant added. In group 5 (animals from 1656 to1659) plasmid pcDNA-Gag+pcDNA-LR1C1_EEO_ (250μg/animal each) and animals were boosted twice with dGag-LR1C1 VLPs with no adjuvant added.
(PPTX)

S1 Table Primer sequences used for in vitro random mutagenesis, nested RT-PCR HIV env amplification and sequencing (standard and deep-sequencing). (DOCX)

S2 Table Neutralization profile of AC10_29 recombinant virus (Clade B, tier 2) used as template for generating the virion library. (DOCX)

S3 Table Comparison of mutation percentages within the same envelope regions (amplicons) in standard and deep-sequencing before and after selection. (DOCX)
