# Supplementary figures and images for: Guiding the humoral response against HIV-1 toward a MPER adjacent region by immunization with a VLP-formulated antibody-selected envelope variant
Source: PLoS One. 2018 Dec 19;13(12):e0208345. doi: 10.1371/journal.pone.0208345 (PMC6300218; doi:10.1371/journal.pone.0208345)

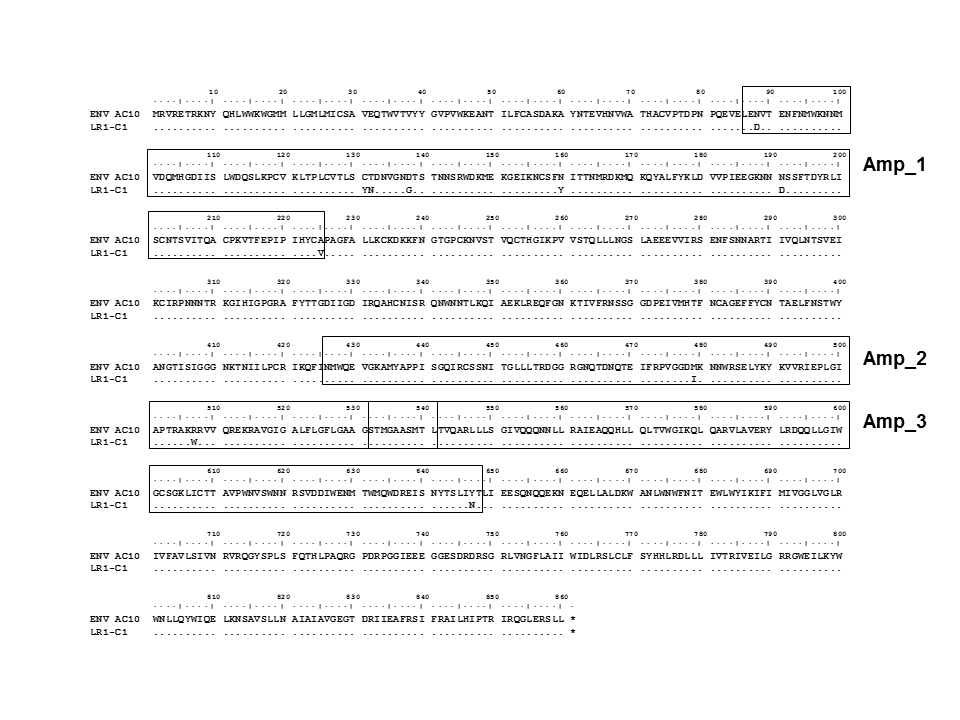

Supplement: S1 Fig — Deep-sequencing of the different Env variants was achieved by sequencing of 3 PCR amplicons (Amp_1, Amp_2 and Amp_3) covering regions of interest. (TIF) [file pone.0208345.s001.tif]

## Slide 1
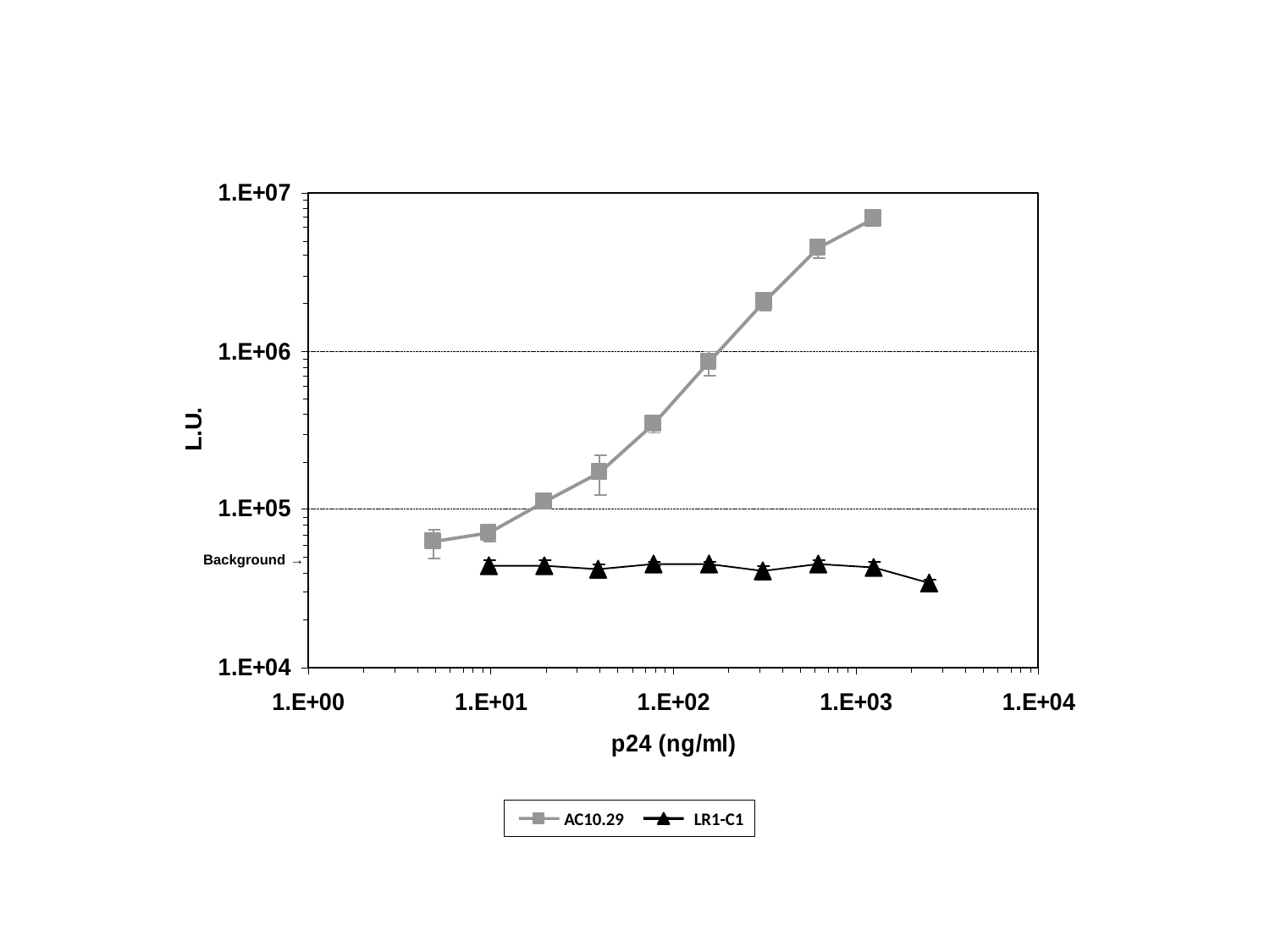

Background →
AC10.29
LR1-C1

Supplement: S2 Fig — Virus stocks were obtained from the transfection of 293T cells. Stocks were normalized to the amount of p24 and used to infect TZM-bl cells. Luciferase activity (L.U.) was measured as described previously [39]. (PPTX) [file pone.0208345.s002.pptx]

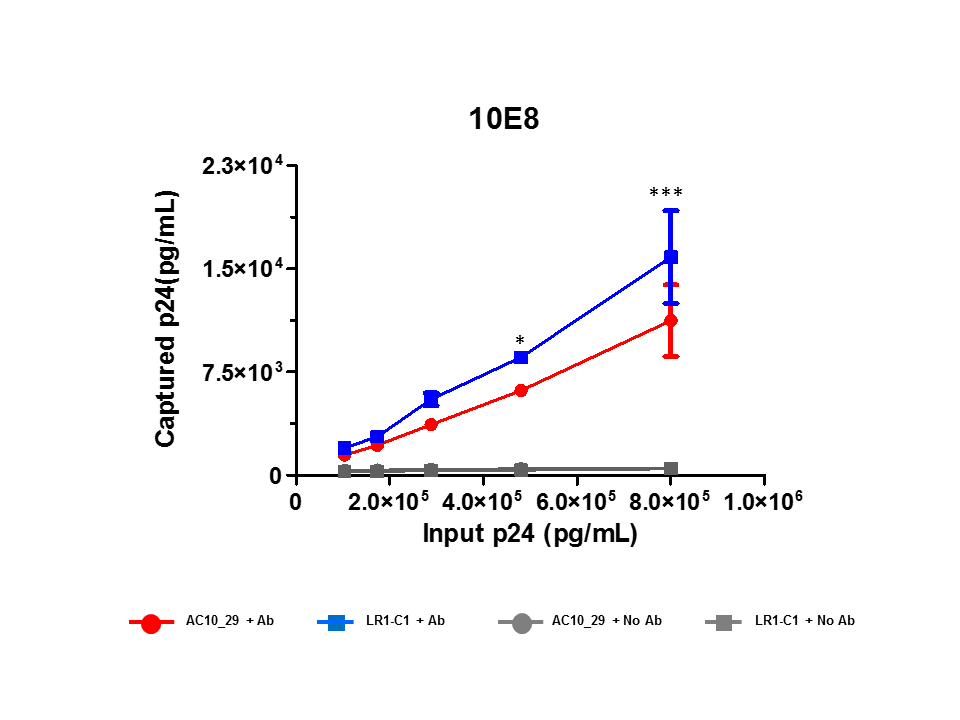

Supplement: S3 Fig — Virus stocks were obtained from transfected 293T cells. Increase in 10E8 binding was determined by comparison of the virus captured by the antibody and quantified by p24 when similar amounts of virus (AC10_29 in red and filled circles and LR1-C1 in blue and filled squares) were used as input in a Virion Capture Assay (VCA). Similar amounts of virus suspension with no mAb were used as controls for both viruses (AC10_29 in grey and filled circles and LR1-C1 in grey and filled squares). Statistical analysis was conducted by R using One-way NOVA followed by Newman Keuls post hoc test at each input concentration (***p<0.001 and *p<0.05). Data are represented as mean ± SDEV. (TIF) [file pone.0208345.s003.tif]
