# Supplementary material for: Guiding the humoral response against HIV-1 toward a MPER adjacent region by immunization with a VLP-formulated antibody-selected envelope variant
Source: PLoS One. 2018 Dec 19;13(12):e0208345. doi: 10.1371/journal.pone.0208345 (PMC6300218; doi:10.1371/journal.pone.0208345)
Supplement: S4 Fig — Balb/c mice were inoculated at weeks 0 and 2 and bled at week 4. Five animals per group were used. Five prime-boost immunization protocols were followed. Both groups were inoculated with 100μl of AT-2 inactivated virus at weeks 0 and 2 (2.5μg/ml p24 quantified as explained in the Materials and Methods section) and 100μl of complete Freund adjuvant subcutaneously). Group 1 was inoculated with AC10 AT-2 inactivated virions and group 2 was inoculated with LR1-C1 AT-2 inactivated virions. At week 4, animals were bled by cardiac puncture with anesthesia from both groups. These experiments were performed at BSL3 CReSA Biocontainment Facility with the collaboration of CReSA staff (Barcelona, Spain). (PPTX) [file pone.0208345.s004.pptx]

## Slide 1
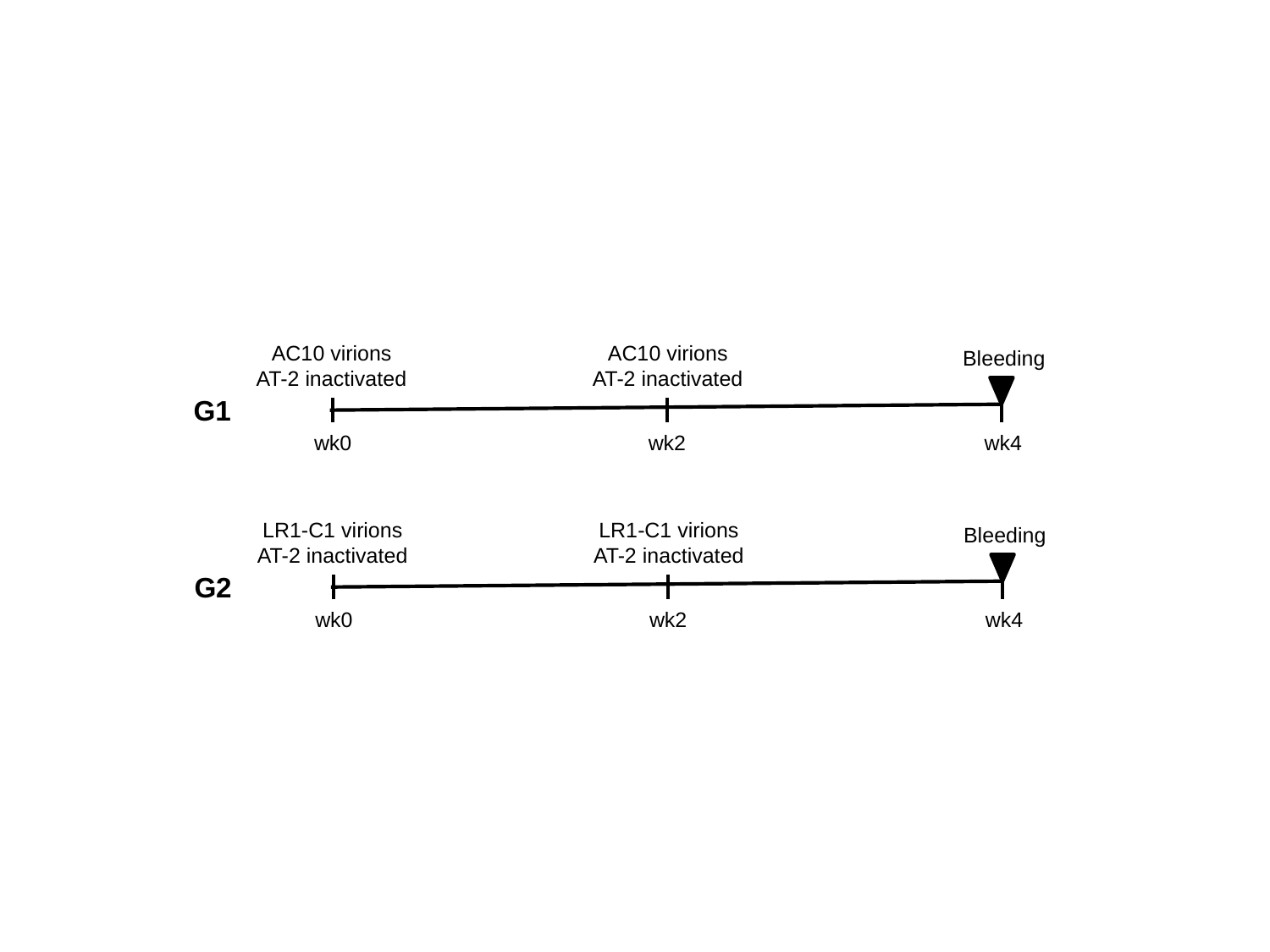

AC10 virions
AT-2 inactivated
AC10 virions
AT-2 inactivated
Bleeding
G1
wk0
wk2
wk4
LR1-C1 virions
AT-2 inactivated
LR1-C1 virions
AT-2 inactivated
Bleeding
G2
wk0
wk2
wk4
