# Supplementary material for: Guiding the humoral response against HIV-1 toward a MPER adjacent region by immunization with a VLP-formulated antibody-selected envelope variant
Source: PLoS One. 2018 Dec 19;13(12):e0208345. doi: 10.1371/journal.pone.0208345 (PMC6300218; doi:10.1371/journal.pone.0208345)
Supplement: S5 Fig — Animals were inoculated at weeks 0, 3, 6, 9, 12 and bled at week 15. Four animals per group were used. Five prime-boost immunization protocols were followed. In group 1 (animals from 1640 to 1643) plasmid pcDNA-Gag+pcDNA (250μg/animal each) were boosted twice with dGag VLPs using adjuplex as adjuvant. In group 2 (animals from 1644 to 1647) plasmid pcDNA-Gag+pcDNA-AC10EEO (250μg/boost/animal each) and animals were boosted twice with dGag-AC10 VLPs (20μg of p24/boost/animal) using adjuplex as adjuvant. In group 3 (animals from 1648 to 1651) plasmid pcDNA-Gag+pcDNA-LR1C1EEO (250μg/animal each) and animals were boosted twice with dGag LR1C1 VLPs using adjuplex as adjuvant. In group 4 (animals from 1652 to 1655) plasmid pcDNA-Gag+pcDNA-AC10EEO (250μg/animal each) and animals were boosted twice with dGag-AC10 VLPs with no adjuvant added. In group 5 (animals from 1656 to1659) plasmid pcDNA-Gag+pcDNA-LR1C1EEO (250μg/animal each) and animals were boosted twice with dGag-LR1C1 VLPs with no adjuvant added. (PPTX) [file pone.0208345.s005.pptx]

## Slide 1
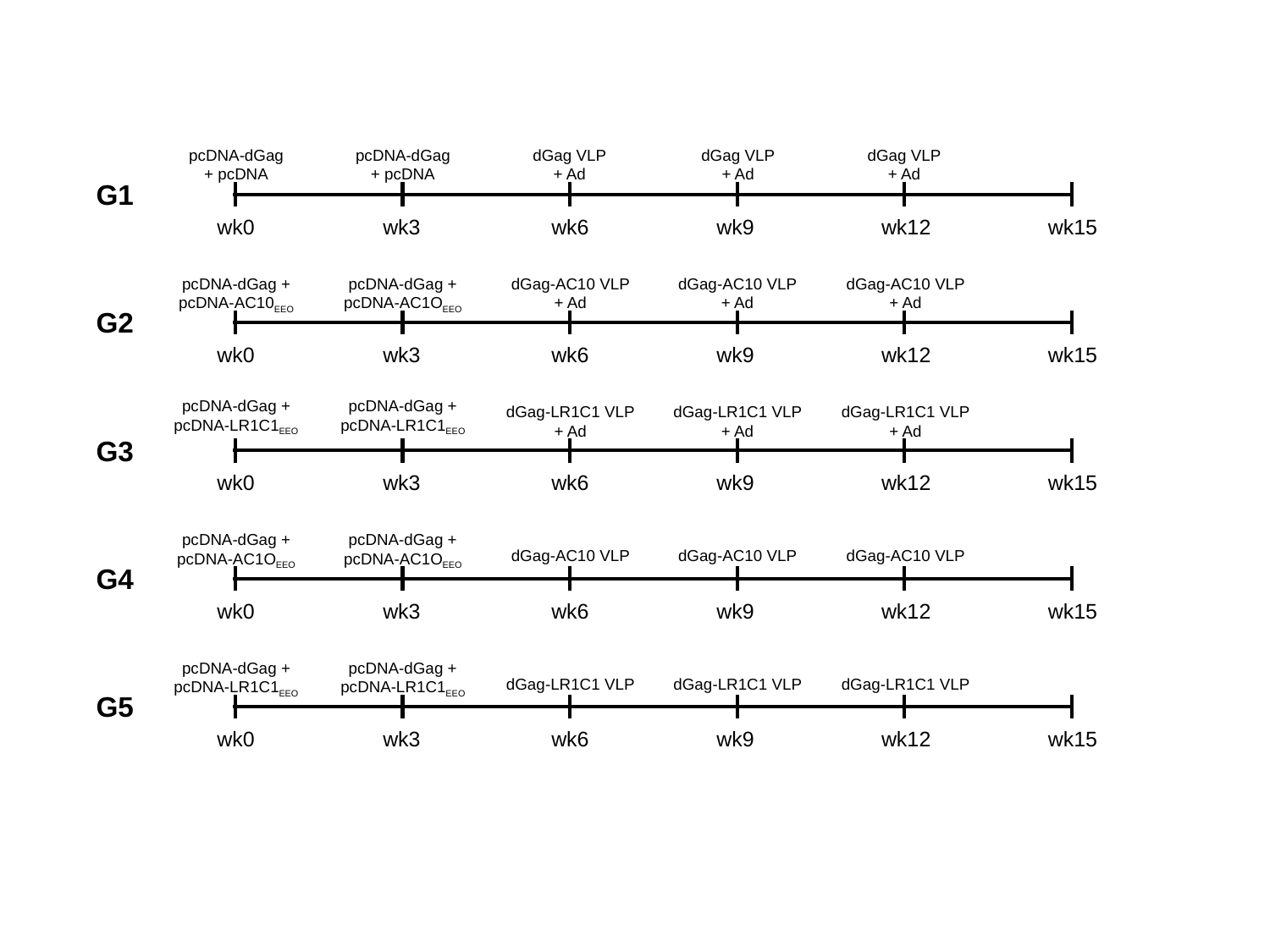

pcDNA-dGag
+ pcDNA
pcDNA-dGag
+ pcDNA
dGag VLP
+ Ad
dGag VLP
+ Ad
dGag VLP
+ Ad
G1
wk0
wk3
wk6
wk9
wk12
wk15
pcDNA-dGag +
pcDNA-AC10EEO
pcDNA-dGag +
pcDNA-AC1OEEO
dGag-AC10 VLP
+ Ad
dGag-AC10 VLP
+ Ad
dGag-AC10 VLP
+ Ad
G2
wk0
wk3
wk6
wk9
wk12
wk15
pcDNA-dGag +
pcDNA-LR1C1EEO
pcDNA-dGag +
pcDNA-LR1C1EEO
dGag-LR1C1 VLP
+ Ad
dGag-LR1C1 VLP
+ Ad
dGag-LR1C1 VLP
+ Ad
G3
wk0
wk3
wk6
wk9
wk12
wk15
pcDNA-dGag +
pcDNA-AC1OEEO
pcDNA-dGag +
pcDNA-AC1OEEO
dGag-AC10 VLP
dGag-AC10 VLP
dGag-AC10 VLP
G4
wk0
wk3
wk6
wk9
wk12
wk15
pcDNA-dGag +
pcDNA-LR1C1EEO
pcDNA-dGag +
pcDNA-LR1C1EEO
dGag-LR1C1 VLP
dGag-LR1C1 VLP
dGag-LR1C1 VLP
G5
wk0
wk3
wk6
wk9
wk12
wk15
