# Supplementary material for: Guiding the humoral response against HIV-1 toward a MPER adjacent region by immunization with a VLP-formulated antibody-selected envelope variant
Source: PLoS One. 2018 Dec 19;13(12):e0208345. doi: 10.1371/journal.pone.0208345 (PMC6300218; doi:10.1371/journal.pone.0208345)
Supplement: S1 Table — (DOCX) [file pone.0208345.s006.docx]

**S1 Table.** Primer sequences used for in vitro random mutagenesis, nested RT-PCR HIV env amplification and sequencing (standard and deep-sequencing).

| **Primer** | **Sequence (5´to 3´)** | **Location^a^** |
| --- | --- | --- |
| 179 | GTAGTACATGTAATGCAACC | 6050 to 6069 |
| 180 | AGCTCGTCTCATTCTTTCCC | 8865 to 8846 |
| 183 | CCAATTCCCATACATTATTGTGC | 6858 to 6880 |
| 185 | GGAGCAGCAGGAAGCACTATGGGC | 7794 to 7817 |
| 186 | GAGTTAGGCAGGGATACTCACC | 8344 to 8365 |
| 190 | GCCAGGACTCTTGCCTGGAGCTG | 7969 to 7947 |
| 192 | CTTGTATTGTTGTTGGGTC | 7135 to 7117 |
| 193 | CATGGCTTTAGGCTTTGATCCC | 6580 to 6559 |
| 102 | TTGCTACTTGTGATTGCTCCATGT | 8936 to 8913 |
| 101 | TAGAGCCCTGGAAGCATCCAGGAAG | 5853 to 5877 |
| 104 | AGCTGGATCCGTCTCGAGATACTGCTCCCACCC | 8916 to 8882 |
| 454_1 | CCCCAACCCACAAGAAGTAG | 6458 to 6477 |
| 454_2 | GCTGGTTTTGCGCTTCTAAAATG | 6885 to 6907 |
| 454_3 | 5´CCCATGCAGAATAAAACAATTTATAA | 7472 to 7497 |
| 454_4 | CTGACGGTACAGGCCAGA | 7833 to 7850 |
| 454_5 | TCTTGGGAGCAGCAGGAA | 7789 to 7806 |
| 454_6 | TTAATTGAAGAATCGCAGAAT | 8157 to 8177 |

^a^Residue numbers correspond to those of the genome of HIV-1 isolate HXB2 (Genebank accession number K03455).
