# Supplementary material for: Guiding the humoral response against HIV-1 toward a MPER adjacent region by immunization with a VLP-formulated antibody-selected envelope variant
Source: PLoS One. 2018 Dec 19;13(12):e0208345. doi: 10.1371/journal.pone.0208345 (PMC6300218; doi:10.1371/journal.pone.0208345)
Supplement: S2 Table — (DOCX) [file pone.0208345.s007.docx]

**S2 Table.** Neutralization profile of AC10_29 recombinant virus (Clade B, tier 2) used as template for generating the virion library.

|  | IC_50_ [µg/ml] | CATNAP profile  IC_50_ [µg/ml]* |
| --- | --- | --- |
| 4E10 | 0.4 | 0.37 |
| 2F5 | 2 | 0.90 |
| 5F3 | >10 | n.d. |
| VRC01 | 5 | 1.46 |
| 2G12 | >12.5 | >50 |
| 447-52D | >10 | >50 |
| PGT151 | 0.006 | 0.005 |
| PG16 | 0.02 | 0.01 |

*Geometric mean of all the data available at the CATNAP database (<http://hiv.lanl.gov/catnap>) [31]

n.d. No data available.
