# Supplementary material for: Guiding the humoral response against HIV-1 toward a MPER adjacent region by immunization with a VLP-formulated antibody-selected envelope variant
Source: PLoS One. 2018 Dec 19;13(12):e0208345. doi: 10.1371/journal.pone.0208345 (PMC6300218; doi:10.1371/journal.pone.0208345)
Supplement: S3 Table — (DOCX) [file pone.0208345.s008.docx]

**S3 Table.** Comparison of mutation percentages within the same envelope regions (amplicons) in standard and deep-sequencing before and after selection

|  | Mutations | | | | | | | | | | | | | |
| --- | --- | --- | --- | --- | --- | --- | --- | --- | --- | --- | --- | --- | --- | --- |
|  | G→A | G→T | G→C | A→T | A→G | A→C | T→C | T→A | T→G | C→A | C→T | C→G | Del.^c^ | Ins.^d^ |
| **L (Standard seq.; %)^a^** | **23.5** | **6.1** | **3.1** | **14.3** | **9.2** | **1.0** | **6.1** | **13.3** | **6.1** | **6.1** | **6.1** | **3.1** | **2.0** | **0.0** |
| L (Deep-seq.; %)^b^ | 19.4 | 6.6 | 1.4 | 12.1 | 6.4 | 1.6 | 11.6 | 11.7 | 0.0 | 3.7 | 21.1 | 3.4 | 0.9 | 0.0 |
| **4E10sL (Standard seq.; %)^a^** | **24.5** | **1.9** | **3.8** | **13.2** | **24.5** | **0.0** | **1.9** | **7.5** | **3.8** | **15.1** | **3.8** | **0.0** | **0.0** | **0.0** |
| 4E10sL (Deep-seq.; %)^b^ | 13.9 | 5.6 | 6.3 | 21.4 | 19.1 | 3.2 | 7.0 | 12.7 | 1.8 | 2.5 | 5.8 | 0.7 | 0.0 | 0.0 |

^a^ The *env* nucleotide sequence of 15 clones from the initial library (L) and 7 clones from the library after one round of R.I.S. selection with 4E10 monoclonal antibody (4E10sL) was determined as described in Materials and Methods section. The percentages are calculated over the total number of mutations.

^b^ More than 9,000 sequence reads were obtained for each of the 3 amplicons generated (Supplementary Fig. 1) from both the initial library (L) and the library after one round of R.I.S. selection with 4E10 monoclonal antibody as described in Materials and Methods section. The percentages are calculated over the total number of mutations.

^c^ Nucleotide deletions

^d^ Nucleotide insertions.
